# Modeling and analysis of biomagnetic blood Carreau fluid flow through a stenosis artery with magnetic heat transfer: A transient study

**DOI:** 10.1371/journal.pone.0192138

**Published:** 2018-02-28

**Authors:** Mohammad Yaghoub Abdollahzadeh Jamalabadi, Mohammadreza Daqiqshirazi, Hossein Nasiri, Mohammad Reza Safaei, Truong Khang Nguyen

**Affiliations:** 1 Department of Mechanical, Robotics and Energy Engineering, Dongguk University, Seoul, Republic of Korea; 2 School of Mechanical Engineering, College of Engineering, University of Tehran, Tehran, Iran; 3 Department of Mechanical Engineering, Daneshpajoohan Higher Education Institute, Isfahan, Iran; 4 Division of Computational Physics, Institute for Computational Science, Ton Duc Thang University, Ho Chi Minh City, Vietnam; 5 Faculty of Electrical and Electronics Engineering, Ton Duc Thang University, Ho Chi Minh City, Vietnam; Worcester Polytechnic Institute, UNITED STATES

## Abstract

We present a numerical investigation of tapered arteries that addresses the transient simulation of non-Newtonian bio-magnetic fluid dynamics (BFD) of blood through a stenosis artery in the presence of a transverse magnetic field. The current model is consistent with ferro-hydrodynamic (FHD) and magneto-hydrodynamic (MHD) principles. In the present work, blood in small arteries is analyzed using the Carreau-Yasuda model. The arterial wall is assumed to be fixed with cosine geometry for the stenosis. A parametric study was conducted to reveal the effects of the stenosis intensity and the Hartman number on a wide range of flow parameters, such as the flow velocity, temperature, and wall shear stress. Current findings are in a good agreement with recent findings in previous research studies. The results show that wall temperature control can keep the blood in its ideal blood temperature range (below 40°C) and that a severe pressure drop occurs for blockages of more than 60 percent. Additionally, with an increase in the Ha number, a velocity drop in the blood vessel is experienced.

## Introduction

Fluid flow that contains bio-magnetic materials is an interesting field of study for bio-engineers [1–4]. Because of the properties of bio-magnetic materials, it is possible to probe the dynamic characteristics of biological fluids that are exposed to an external magnetic field [5–8]. The occlusion of arteries (narrowing of the coronary) and blood vessels is one of the most serious problems of humanity that is faced in our era [9–11]. In such geometries, the flow conditions and blood characteristics have a major effect on the flow pattern, which by themselves can lead to a stent rupture and cause an embolism. In fact, the blood movement and mechanical behavior of the vessel walls are known for their key role in the formation of blood vessel stenosis [12].

Therefore, the importance of blood rheology, to evaluate the desired flow variables, cannot be ignored. Among other factors, the shear stress has a significant role in thrombosis and the development of pathological aneurysms. Concerning this problem, earlier studies [13–15] have considered blood mainly as a Newtonian fluid. Even though this model is a simplified form of the general case, it conserves some basic aspects of the flow, especially in the larger arteries. For example, Tanwar [16] analytically investigated the effect of a magnetic field on Newtonian blood flows to understand the abnormal flow conditions of blood in a locally constricted blood vessel. Reviews of flow patterns and research on stenotic arteries can be found in [17, 18]. Blood flow under surgical conditions or diagnosis operations have been considered in a few publications, for example, Srivastava accounted for the catheterization of operations [19].

Other studies [20, 21] have examined the influence of non-Newtonian blood flows. This improved model has revealed new results that were more realistic. It is common to see such flow patterns and non-Newtonian behaviors manifest themselves in small blood vessels. Haldar [22] investigated the shape of a mild stenosis; this author declared that shear thinning is a more dominant non-Newtonian behavior in comparison to viscoelasticity. In some other studies, the pulsatile pressure was the focus of the study. A broad range of formulations and boundary conditions were utilized and studied in these analyses. Of these, the following notable aspects were considered: irregular stenosis with the development of a generalized power law [23], weak-form of the Casson equation [24], finite element formulation [25], overlapped stenosis [26], and the Lattice Boltzmann Method [27]. In another study, blood was represented by two layers, with each layer demonstrating a different viscosity behavior (i.e., the combined Newtonian and generalized power law) [28]. Lukacova [29] considered the fluid structure interaction of stenosed vessels, wherein the Carreau model was used to represent the blood properties. The shapes of the aortic arteries were studied by Lie *et al*. [30], to research the pressure drop as well as the wall shear stress. The effect of unsteadiness of the non-Newtonian micropolar fluid was investigated through an analytical solution with regard to the modified Bessel function in [31, 32]. Significant rheological impacts were reportsed in these studies. Therefore, an emphasis must be placed on non-Newtonian modeling in any small vessel blood analysis.

Studies related to magnetization are present in another collection of investigations [33–41]. These studies conclude that magnetization has a profound effect on both the blood circulation and the blood pressure drop. Among others, Tzirtzilakis [33] studied bio-magnetic channel flows. Ikbal [34] investigated stenosed vessels under the influence of transverse magnetization. Varshney [35] studied the occurrence of multiple stenoses in a blood vessel. Multiple aneurysms under magnetization was studied for the purpose of understanding flow patterns [36]. Behnia *et al*. studied the dynamics of a bubble in non-Newtonian blood [37] and under magnetization [38]. Alimohammadi *et al*. [39] considered carotid bifurcation in the presence of porous plaques and vessel walls under magnetization. The entropy generation of the magneto-hydrodynamics problem of blood flow is found in [40] for nanotubes and in [41] for a case with permeable walls.

When blood receives magnetic heating, its heat will pass through the whole vessel, from the heated zone to the end of the blood vessel [42]. In the presence of magnetization, secondary circulation is induced that originates from the Joule heating [43]. Such a source of heat has been proven to decrease pain, inflammation, and swelling, which is considered to be relaxing the muscle and nerves. It has even been suggested to enhance circulation by removing circulatory blockages [44] or by softening small-scale lumps in patients [45]. Nonetheless, it could be fatal when blood under magnetization starts to degrade, at temperatures above forty degrees.

The rise in the number of breast tumor, uterus tumor, and lymph tumor diagnoses has encouraged some researchers to foster the idea of heat therapy as an instrument for the prevention of tumors and cancers [46]. Many papers on heat therapy applications exist in the literature. For example, heat therapy was effectively used in intervertebral discs [47], dysmenorrhea syndrome [48], chest pain [49], and neck pain [50]. Magnetic therapy and blood vessels dilated with the use of its induced heat can be used as an alternative method for cancer therapy and other medical treatments [45]. In these medical functions, the issue of vessel wall cooling is proposed as an efficient approach to sustaining the living conditions of the blood.

The literature survey suggests that there is a gap for a study of an essential case of stenosis in which blood is assumed to be non-Newtonian and the vessel is subjected to magnetization, especially with considering the induced heat, the heat transfer phenomena, and the paramountcy of the blood vessel temperature control and cooling. As the real phenomenon that occurs in transient form, such a case in a transient manner is studied in our research.

## Mathematical modeling

This study uses a mathematical model of a bio-magnetic fluid and employs the Carreau formulation for representing the non-Newtonian behavior in the model. The developed model consists of both ferro-hydrodynamic (FHD) and magneto-hydrodynamics (MHD) principles, where the blood conductivity and magnetization are considered. A transient method is used to solve the governing equations on a single grid. Blood is an important bio-magnetic fluid that demonstrates magnetic fluid behavior that is governed by the relationship between the intercellular protein, cell membrane and hemoglobin, which is a form of iron oxide that is present in the mature red blood cells extensively. It should be noted that the status and formulations of blood magnetic properties are in a close association with the oxygenation content [42]. Therefore, the nonlinear governing equations for such a transient, incompressible and laminar flow are the conservation of mass [51]

∇⋅V=0 (1)

the momentum [51]

$$\frac{\rho DV}{Dt}=-\nabla p+\rho F+\mu \nabla^{2}V+J\times B+\mu_{0}M\nabla H$$ (2)

and the energy [43]:

$$\rho C_{p}\frac{DT}{Dt}+\mu_{0}T\frac{\partial M}{\partial T}\frac{DH}{Dt}-\frac{J\cdot J}{\sigma }=k\;\nabla^{2}T+\mu \Phi$$ (3)

In the above formula, **V** is the blood velocity, $\frac{D}{Dt}=\frac{\partial }{\partial t}+V\nabla$ is the material time derivative, *ρ* is the fluid density, F is the body force per unit volume, *μ* is the dynamic viscosity, *μ*_0_ is the viscosity at the zero shear rate, **M** is the magnetization vector, **B** is the magnetic induction, **H** is the magnetic field intensity, *σ* is the electrical conductivity of the bio-fluid, **J** is the electric current density, T is the bio-fluid temperature, k is the bio-fluid thermal conductivity, C_p_ is the bio-fluid specific heat (at a constant pressure) and Φ is the viscous dissipation, which in the Cylindrical coordinate system is the following [43]:

$$\Phi_{v}=2\left[{\left(\frac{\partial u_{r}}{\partial r}\right)}^{2}+{\left(\frac{1}{r}\frac{\partial u_{\theta }}{\partial \theta }\frac{u_{r}}{r}\right)}^{2}+{\left(\frac{\partial u_{z}}{\partial z}\right)}^{2}\right]\begin{array}{c} +{\left[r\frac{\partial }{\partial r}(\frac{u_{\theta }}{r})+\frac{1}{r}\frac{\partial u_{r}}{\partial \theta }\right]}^{2}\\ +{\left[\frac{1}{r}\frac{\partial u_{z}}{\partial \theta }+\frac{\partial u_{\theta }}{\partial z}\right]}^{2}+(\frac{\partial u_{r}}{\partial z}+\frac{\partial u_{z}}{\partial r})-\frac{2}{3}{\left(\nabla \cdot V\right)}^{2} \end{array}$$ (4)

Additionally, the viscosity *μ* is shear dependent and can be written as follows [52]:

$$\mu (\dot{\gamma })=\mu_{inf}+(\mu_{1}-\mu_{inf}){\left(1+{\left(\lambda \;\dot{\gamma }\right)}^{2}\right)}^{\frac{n-1}{2}}$$ (5)

In this model, to model the blood correctly, the following values are used: for a zero shear rate limit: (*μ*_1_ = 0.056 Pa.s), infinite shear rate limit: (*μ*_*inf*_ = 0.036 Pa.s), relaxation time constant: (*λ* = 3.313 *s*), and power index: (*n* = 0.3568).

Finally, the governing equations are coupled with magnetic field equations [43]:

∇×H=J=σ(V×B) (6)

$$\dot{\nabla }\cdot B=\nabla \cdot (H+M)=0$$ (7)

It is noted that Eq (1) resembles the balance between the inward and outward mass flows in a steady system. Consequently, this approach dictates that the total entering mass to the system must be equal to the exiting mass. Energy conservation states that energy roughly changes from one form to another. In other words, the energy in a closed system enters or exits it only from its inlets and outlets in the shape of energy flux (the energy measured per unit of cross-sectional area). However, energy cannot be created or destroyed. For example, in the case of heat transfer, heat can be transformed from another kind of energy to it. For example, these conversions can be observed in phenomena such as Joule heating and/or frictional heating. It should be noted that although the magnetization can be regarded as a function of the temperature (for example, look at [53]), in this study, these two parameters are assumed to be independent.

The stenosis geometrical formulation is as follows [19]:

$$\frac{r(z)}{R_{0}}=\left\{ \begin{array}{l} 1-2\frac{\delta }{R_{0}L_{0}}(z-d);\;d<z\leq d+\frac{L_{0}}{2}\\ \;1-\frac{\delta }{2R_{0}}(1+cos\frac{2\pi }{L_{0}}(z-d-\frac{L_{0}}{2}));\;d+\frac{L_{0}}{2}<z\leq d+L_{0}\\ \;1;\;otherwise \end{array}\right.$$ (8)

where *R*_0_ = 0.01 *m* is the radius of the blood vessel in the absence of stenosis. The important constants for this study are summarized in Table 1.

**Table 1:** An overview of the geometrical parameters used in the simulation.

Parameter	Value
Vessel Radius	1 cm
Length	7 cm
Stenosis length	2.824 cm (see Eq 5)
Stenosis height	50 percent of the vessel radius unless declared differently

The boundary conditions are

$$@\;r=R_{0}\;u_{r}=u_{z}=0$$ (9A)

$$@\;r=0\;u_{r}=0,\;\frac{\partial u_{z}}{\partial r}=0$$ (9B)

$$@\;z=l_{0}\;\frac{\partial u_{z}}{\partial z}=0$$ (9C)

@z=0T=300 (9D)

The pulsatile pressure is modeled as a sinusoidal pressure to resemble human blood pressure, which is used in the simulation of real human heart beating, i.e., the pressure is as follows:

$$P(t)=P_{m}\;(1+\epsilon \;sin\;\omega_{0}\;t)$$ (10)

In the above equation, $\epsilon =\frac{P_{s}}{P_{m}}$, where P_m_ is a constant that represents the mean pressure, P_s_ is the amplitude of the sinusoidal pressure, and ω_0_ is the frequency of the oscillation [54]. Some of the simulation constants are listed in Table 2.

**Table 2:** An overview of the simulation constants used in the simulation.

Parameter	Value
C_p_	3.49 $\frac{kJ}{k^{0}C}$
*ρ*	1050 $\frac{Kg}{m^{3}}$
*σ*	0.8 $\frac{S}{m}$

**Fig 1:**
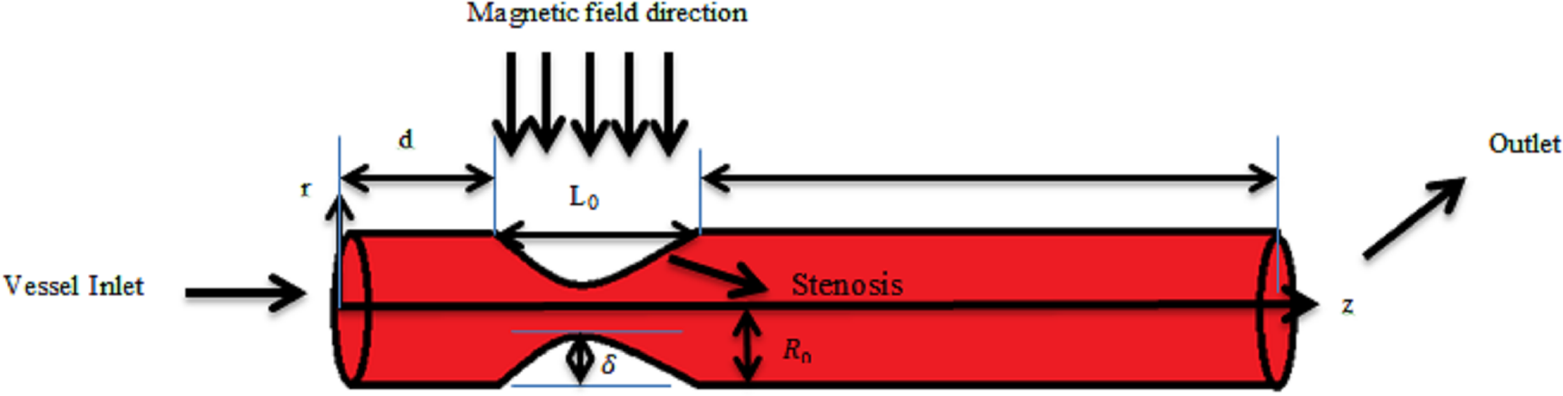
Schematic of the computational domain used in the current work.

As presented in Fig 1, the lowest vessel radius occurs at *z* = 2 cm, where the stenosis ratio is 50 percent of the blood vessel (unless stated otherwise). After the peak in the stenosis, the recirculation and flow properties are highly critical because a decrease in the flow velocities can aggravate the patient’s condition and further enlarge the stenosis by fat accumulations. If the blood is assumed to be Newtonian fluids, the longer vortex would be captured when compared to non-Newtonian fluids. Additionally, because of the lower shear rates inside the recirculation region, higher viscosities could be expected as the flow velocity reduces as a result of the shear thinning effects.

It is worth noting that the Joule heating phenomenon increases the blood temperature, which is paramount, and an action to accomplish its mitigation is imperative. When the blood temperature experiences a 3°C to 4°C hike over its physiological temperature, irreversible damage to the protein in the plasma occurs, and one normally cannot survive after such a high fever [43]. To analyze the effect of the magnetic field and the induced temperature fields, two cases were considered initially. In the first case, non-Newtonian blood is assumed in the blood vessel without imposing a magnetic field. In the second case, the magnetic field was imposed on the second section of the blood vessel. The matter of Joule heating and the employment of a cooling region in the second part of the stenosis are presented graphically.

## Numerical modeling and validation

In this research, the FVM formulation on an unstructured mesh is employed [40]. In the following section, the discretization method is explained. Usually, the FVM method is preferred over other numerical models because it inherently satisfies the continuity law and is easier to implement for unconventional domains.

**Fig 2:**
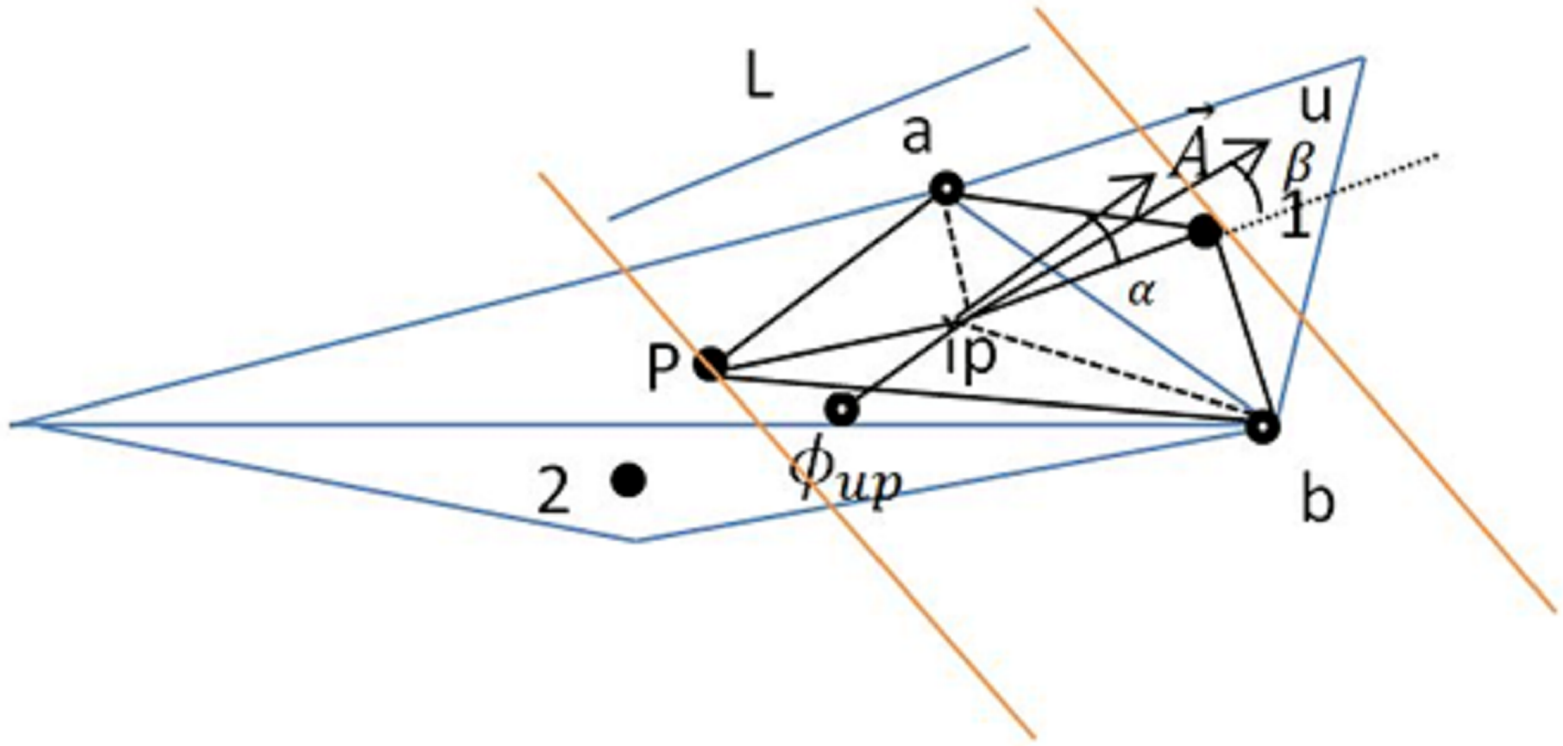
The utilized mesh and control volume for the development of the discretized forms.

For discretization, a staggered grid with a face-normal velocity component is utilized. A mesh and the control volume for discretization are shown in Fig 2.

A general convective-diffusive formulation, where the velocity field is denoted as **V,** for the property of ϕ is

$$\rho \frac{\partial \phi }{\partial t}+V\rho \nabla .\phi =\Gamma^{\phi }\nabla^{2}\phi +S^{\phi }$$ (11)

In the above equation, Γ^ϕ^, *S*^ϕ^,*ρ*, and *t* stand for the diffusion coefficient, source terms, density and time, respectively.

After integration over a volume called P (Fig 2), with the use of the Divergence Theorem with linearized source terms, a numerical integration of space and time is performed. Then, we have the following:

$$M_{p}^{n}\phi_{p}^{n}-M_{p}^{n-1}\phi_{p}^{n-1}+\Delta t{\sum_{ip=1}^{3}\left[\rho (V.A\right)}_{ip}-\Gamma^{\phi }{\left(\nabla \phi_{p}.A\right)}_{ip}-(S_{p}\phi_{ip}+S_{c})\frac{\Delta v_{a1b}}{2}=0$$ (12)

In the above equation, (M_p_ = *ρ*Δ*v*), **A** and Δ*v* are the mass in the control volume, each face area, and the volume of the discrete place, respectively. Additionally, we have [55, 56]

$$\nabla \phi_{p}.A=\frac{\left(x_{1}-x_{p})(y_{0}-y_{b})+(y_{1}-y_{p})(x_{b}-x_{a}\right)}{[{\left(x_{1}-x_{p}\right)}^{2}+{\left(y_{1}-y_{p}\right)}^{2}]{\left(\frac{{(x}_{c}x_{A}+y_{c}y_{a})}{\left(x_{c}^{2}+y_{c}^{2})(x_{A}^{2}+y_{A}^{2}\right)}\right)}^{2}}(\phi_{1}-\phi_{p})$$ (13)

The variables in the above equations are summarized in Table 3.

**Table 3:** Variables in the formulation of the finite volume method in the current formulation.

Variable	Momentum equation	Energy equation
***ϕ***	Any of the velocity components	temperature
**Γ**	Viscosity	$\frac{\kappa }{c_{p}}$
***S***_***P***_	−*σB*.*B*	0
***S***_***c***_	*ρF* + *μ*_0_ *M∇H*	$\mu_{0}T\frac{\partial H}{\partial T}\;\frac{DH}{Dt}+\frac{J.J}{\sigma }+\mu \phi$

The governing equations are transformed into computational space, and then, they are reduced to second-order accurate finite-volume equations. In this study, an FVM formulation with the SIMPLE algorithm [57] is used to discretize the governing equations of the problem; afterward, the transport equation is solved using a time marching procedure. The transient formulation is accounted for in the spatially pulsatile pressure.

**Fig 3:**
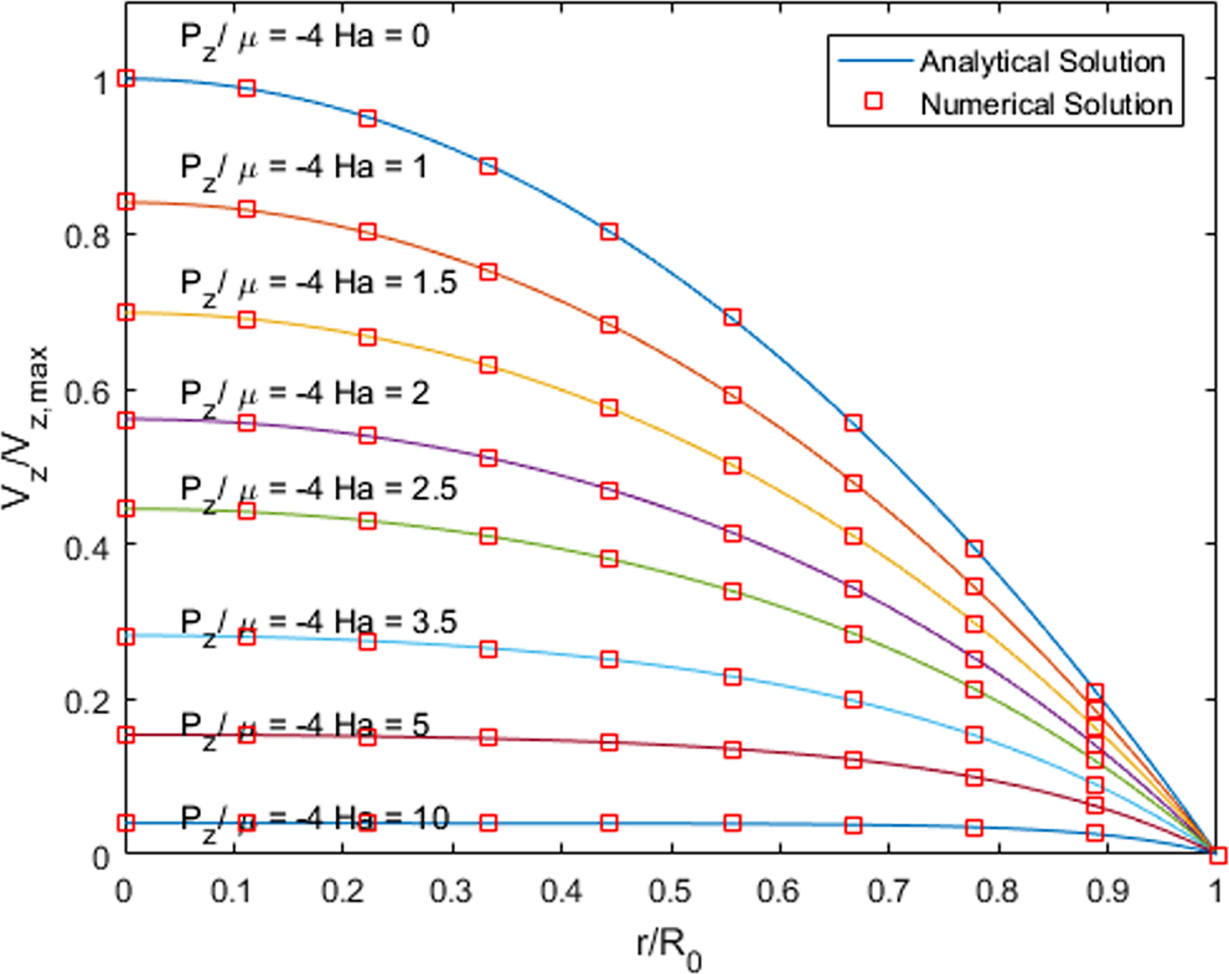
Comparison of the numerical and analytical solution of the pipe flow for different Ha numbers and radii.

Validation of the code was guaranteed through a comparison of the pipe flow with a constant pressure gradient with its analytical solution [58]. Fig 3 represents the non-dimensionalized velocity for different radii with Ha numbers that vary from 0 to 10 and different pressure gradients.

**Fig 4:**
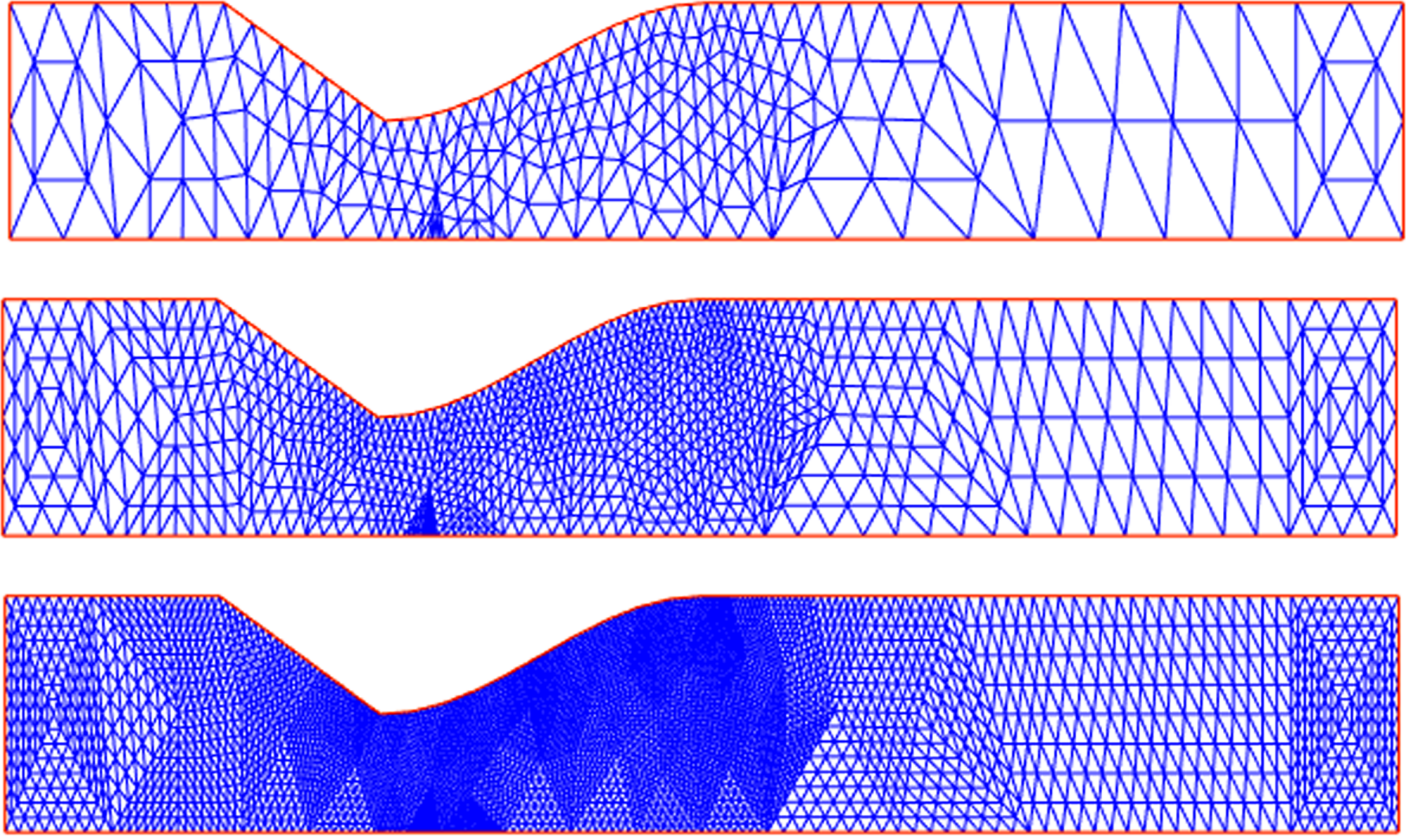
Three different meshes of the mesh independence analysis, with the mesh generation method of Delaunay.

To assure grid independence, four different meshes, with 273, 993, 3777 and 14721 grid points (where the number of nodes increases by an order of two in each direction with the Delaunay triangulation algorithm) were considered in Fig 4 (the three meshes that correspond to 273, 993, and 3777 nodes are presented). The relative errors of the finest mesh for the maximum stress of blood flow in a vessel with a fifty percent stenosis were 38.3131, 14.7454 and 4.6102 percent, respectively. Therefore, the third mesh was taken as the suitable mesh for the study.

## Results and discussion

This section discusses the flow parameters and patterns in a blood vessel in the presence of a stenosis with and without magnetization. Additionally, in one of the cases, the cooling of the vessel wall is examined. Normally, the velocity field and stress are of interest in studies on blood flow in the presence of a stenosis.

Where a magnetization was employed; the blood is under the effect of a constant magnetic field. Moreover, a pulsatile inlet pressure was in the study; therefore, the flow properties cause velocity profile changes with time and position. In this regard, the results and properties in equal periods are presented for comparison.

**Fig 5:**
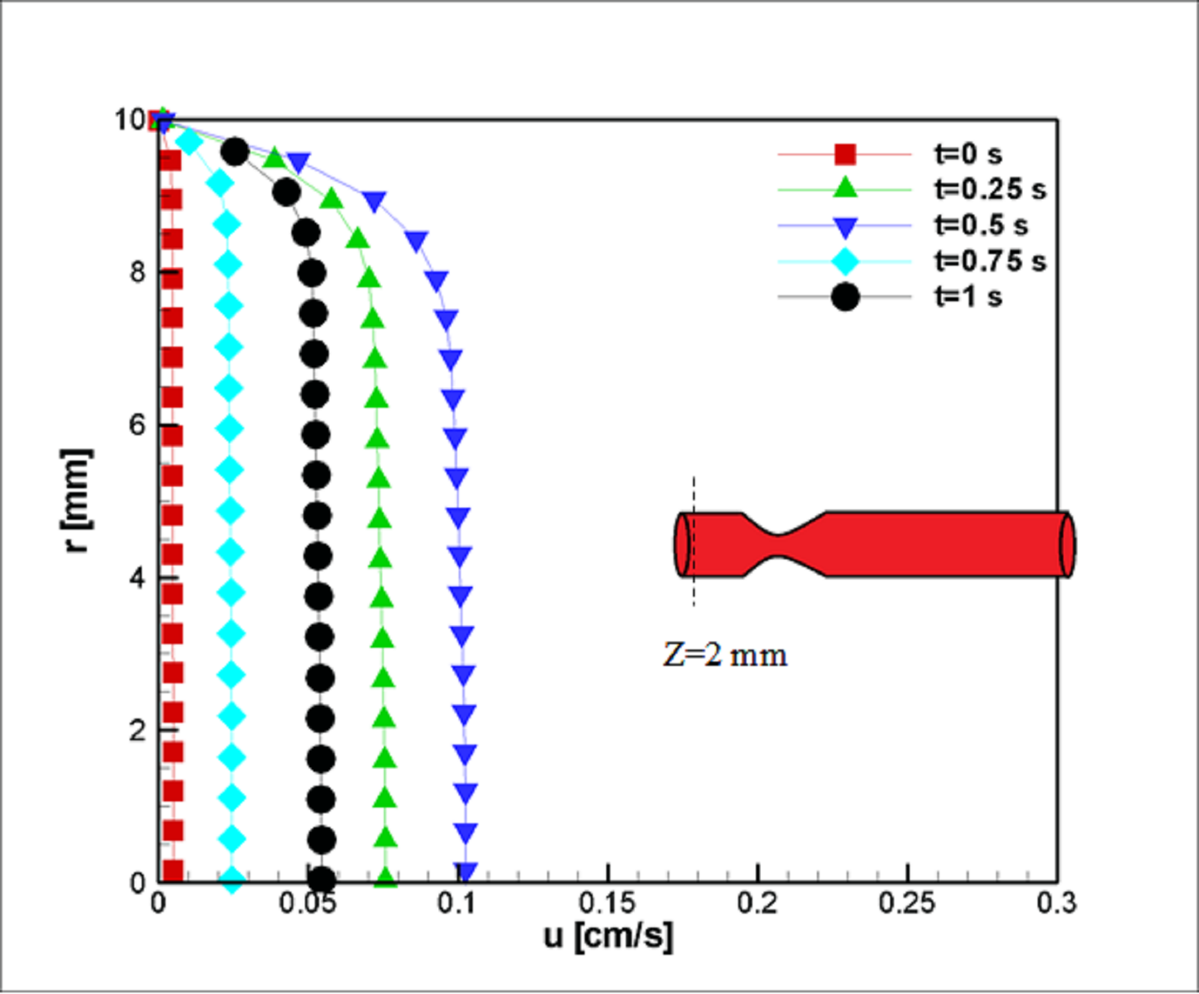
Velocity profile in the axial direction near the inlet at z = 2 mm for the case without Joule heating, *L*_*vessel*_ = 7 *cm*, *R*_*vessel*_ = 1 *cm* and fifty percent stenosis.

Fig 5 depicts the velocity profiles at the vessel inlet, z = 2 mm. This position is too far from the stenosis throat to be influenced by it. Five steps were used to extract the needed data. The velocity field accelerates in the first quarter of the study; it decelerates and reaches a maximum value at half of the time lapses, and then, the velocity outside the boundary layer reduces before undergoing a final increase at the last step. The boundary layer proportionally develops with respect to the flow velocity outside the boundary layer.

**Fig 6:**
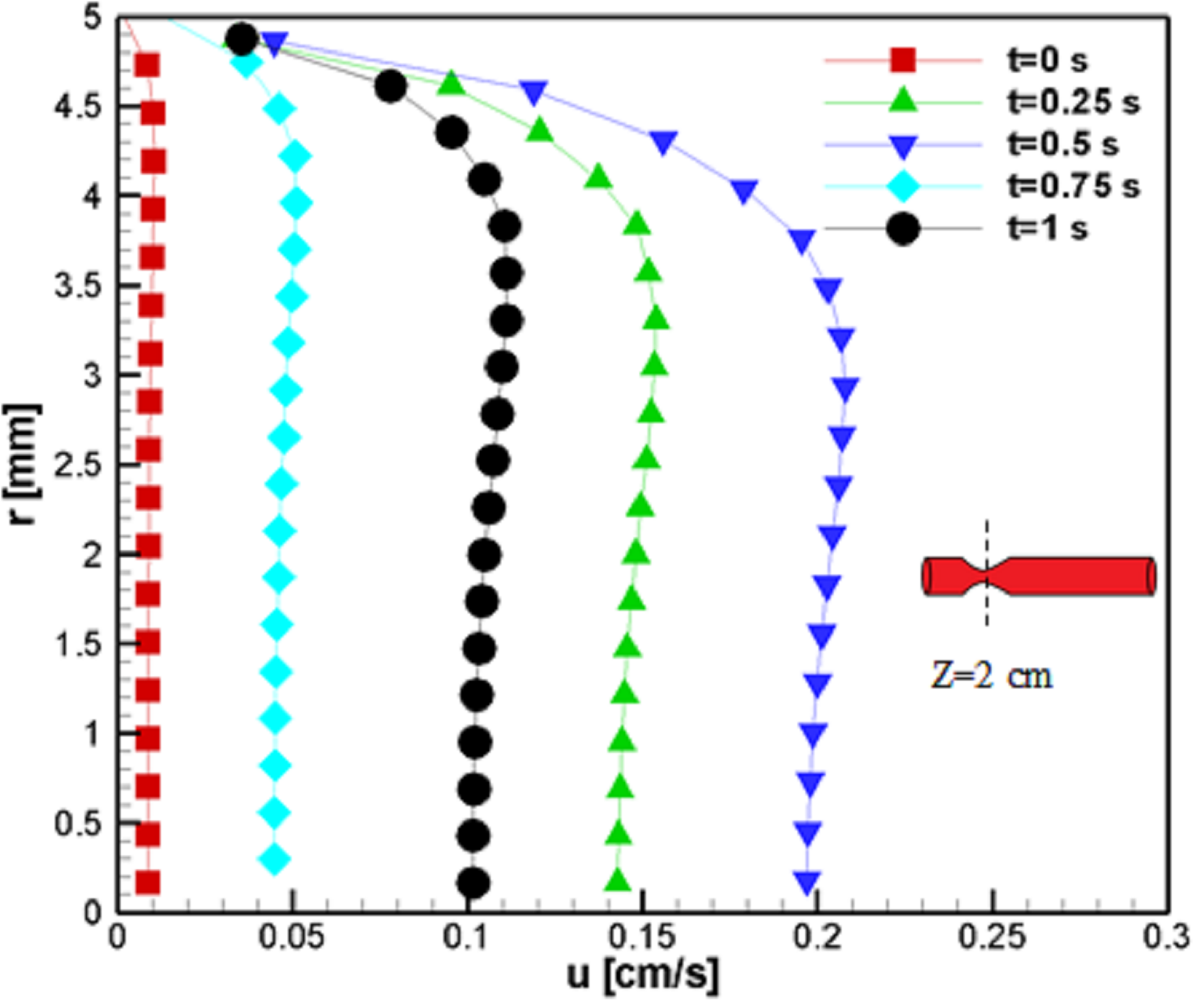
Profile of the axial velocity at z = 2 cm for the case without Joule heating at five different time instances, *L*_*vessel*_ = 7 *cm*, *R*_*vessel*_ = 1 *cm* and fifty percent stenosis.

At the stenosis throat, the temporal development of the flow was quite similar to the inlet condition, although the flow experienced higher and lower velocity values, which suggests higher acceleration and deceleration is needed to conserve continuity. Fig 6 shows the phenomena mentioned above. In addition to this fact, it is evident that the flow velocity reached a maximum value and reduced to a lower value at the blood vessel center; this phenomenon is due to the non-Newtonian behavior of the blood. Furthermore, the velocity peak followed the same trend that occurred at the inlet. Such observation agrees well with velocity profiles in the literature [59].

**Fig 7:**
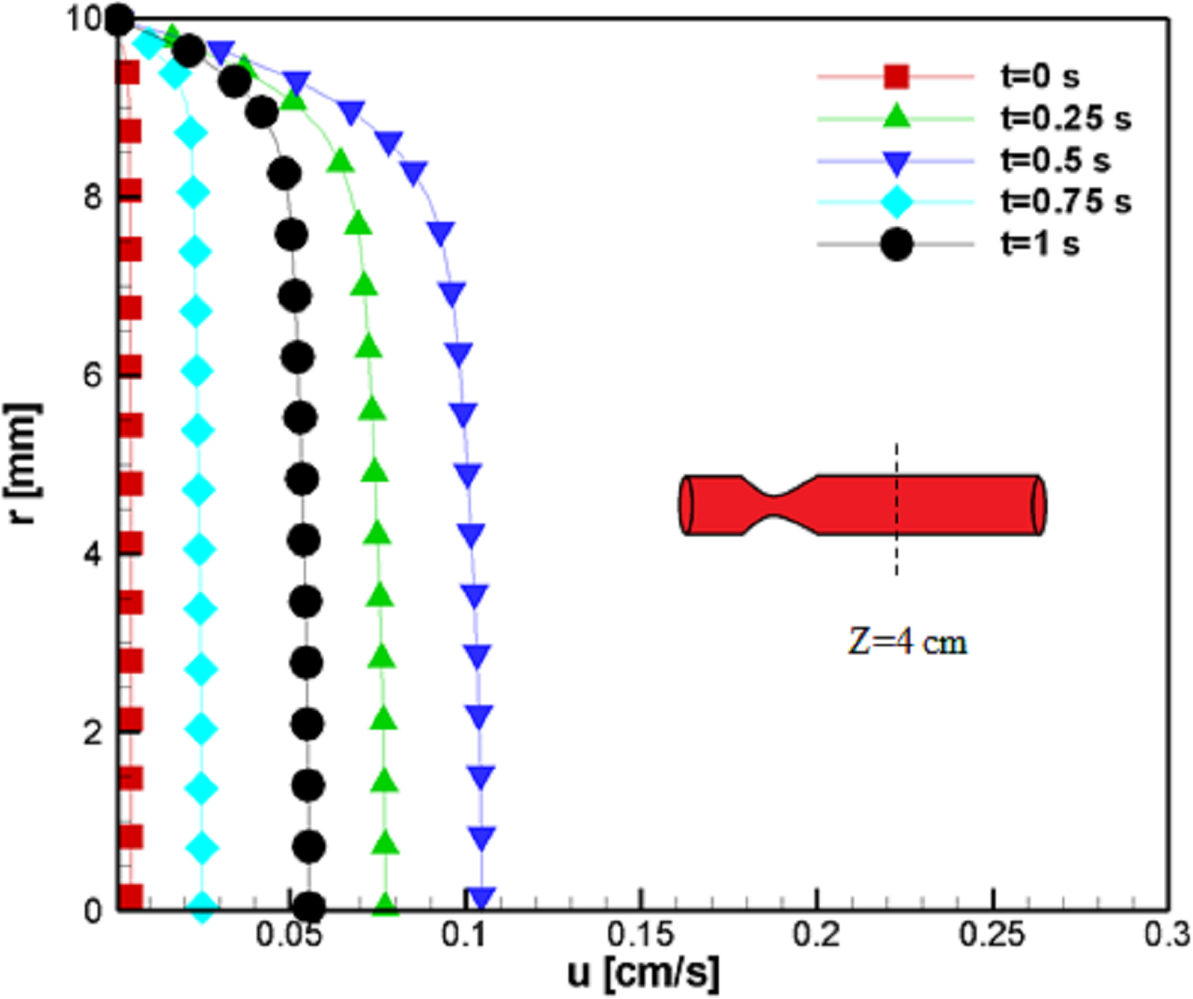
Profile of the velocity in the axial direction at z = 4 cm for the case without Joule heating at five different time instances, *L*_*vessel*_ = 7 *cm*, *R*_*vessel*_ = 1 *cm* and fifty percent stenosis.

To study the effect of the magnetic field and the velocity profiles downstream of the stenosis, we examined the velocity in the direction of the blood vessel axis at *z* = 4 *cm*. The velocity profiles were almost the same as our inlet, which suggests an extreme velocity alteration at all steps (in terms of magnitude). For example, the flow in the middle of the study increases from $u_{z}=0.1\frac{cm}{s}$ to $u_{z}=0.2\frac{cm}{s}$ and decreases to almost the same amount afterward (Fig 7). The reason for such behavior is the variation of the blood pressure pulse during the cardiac cycle. Additionally, this case manifests a lower velocity magnitude, and the findings show that the magnetization lowered the magnitude of the velocity.

**Fig 8:**
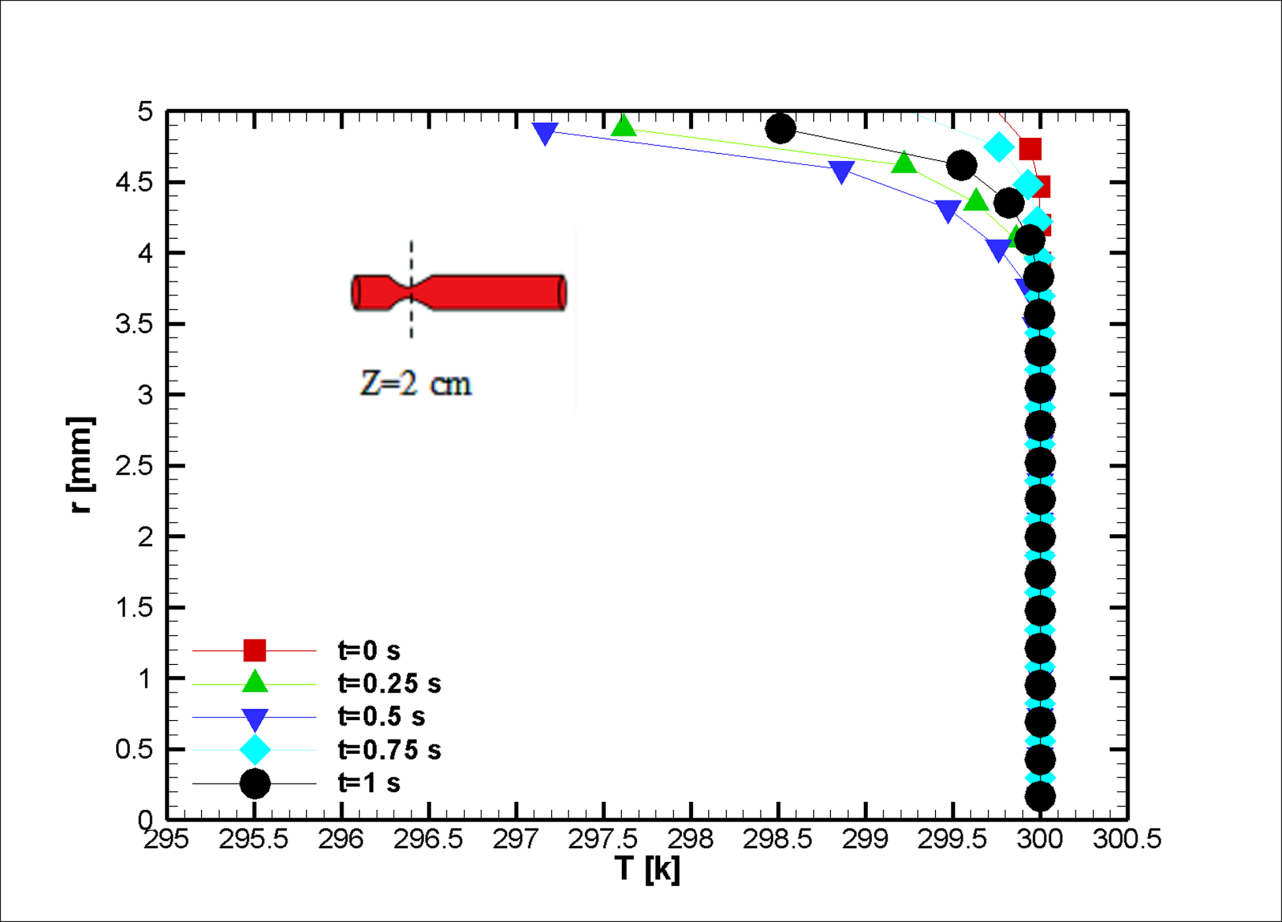
Profile of the temperature distribution at the stenosis at z = 2 cm for the case without Joule heating at five different time instances, *L*_*vessel*_ = 7 *cm*, *R*_*vessel*_ = 1 *cm* and fifty percent stenosis.

**Fig 9:**
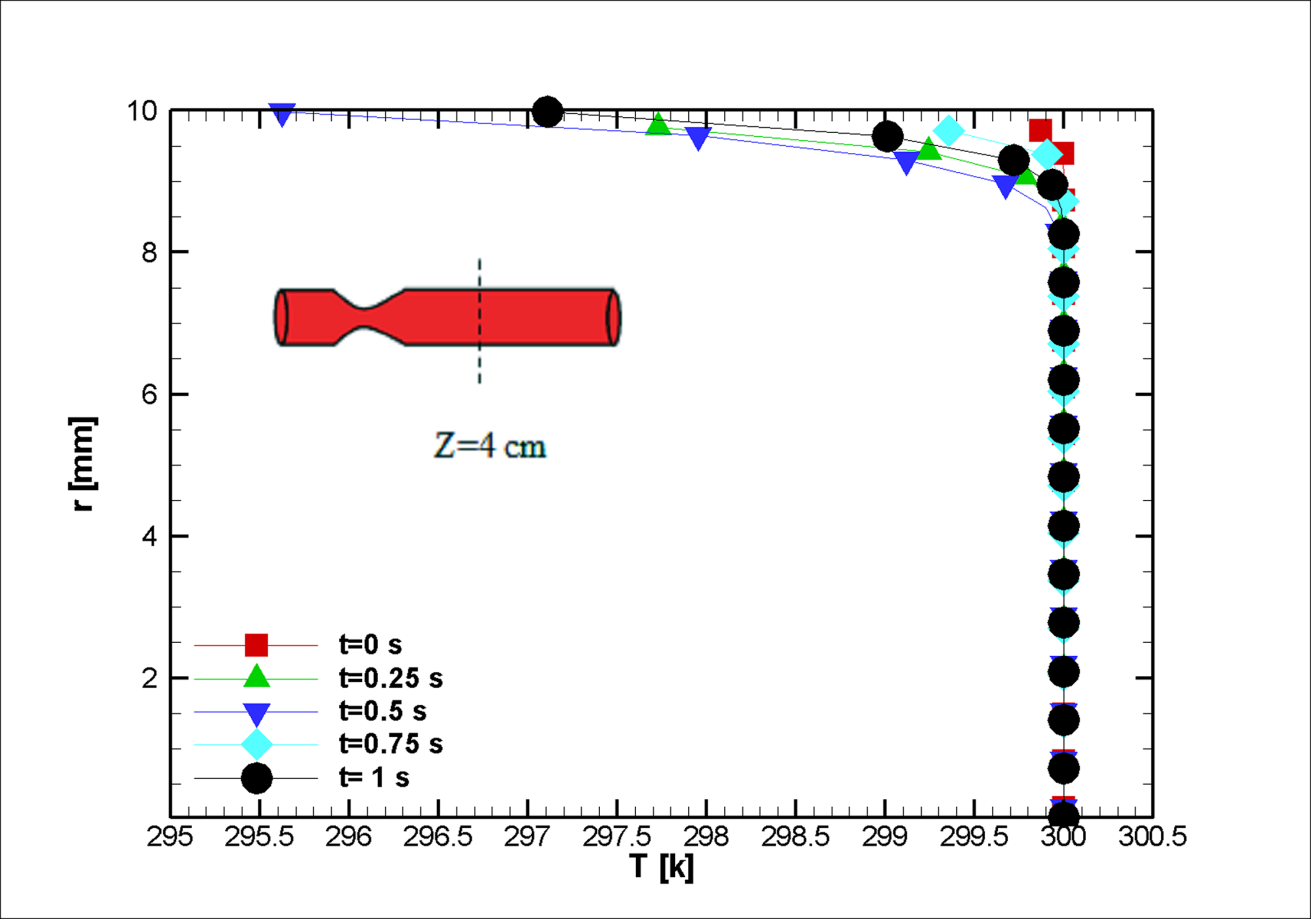
Profile of the temperature distribution at z = 4 cm for the case without Joule heating at five different time instances, *L*_*vessel*_ = 7 *cm*, *R*_*vessel*_ = 1 *cm* and fifty percent stenosis.

When the blood is in motion, its electrical conductivity is always greater than the blood at rest [60]. The greater the volume percentage of the hematocrit (Ht or HTC), the greater the electrical conductivity of the blood [61]. It should be noted that this value for the medium shear rates is approximately 10 percent [23]. In this study, the assumed electrical conductivity of blood equals 0.8 Sm^-1^. Furthermore, the dependence of the electrical conductivity with the temperature is neglected; however, the magnetic behavior of a ferro-fluid is inversely proportional to its temperature (due to Curie’s law). The temperature distribution is shown in Figs 8 and 9. It is worth mentioning that we have surface cooling on stenosis after the stenosis peak. Initially, less variation in temperature is observed; as we reach the halfway point of the simulation, the temperature difference between the wall and the outside of the thermal boundary layer is augmented due to Joule heating, and thus, it can be inferred that the response of the blood to the magnetic field reduces in this region. The change can be informative for drug delivery or other medical procedures where the control of magnetization is essential [62, 63]. Afterward, the blood temperature difference decreases, and up to a third of the simulation has heat dissipated from the cooled wall. Then, it becomes larger in magnitude as the blood velocity reduces. In this case, the blood experiences a mere two degree increase in a small boundary layer. As discussed earlier, keeping the blood at a constant temperature is vital for one’s survival.

**Fig 10:**
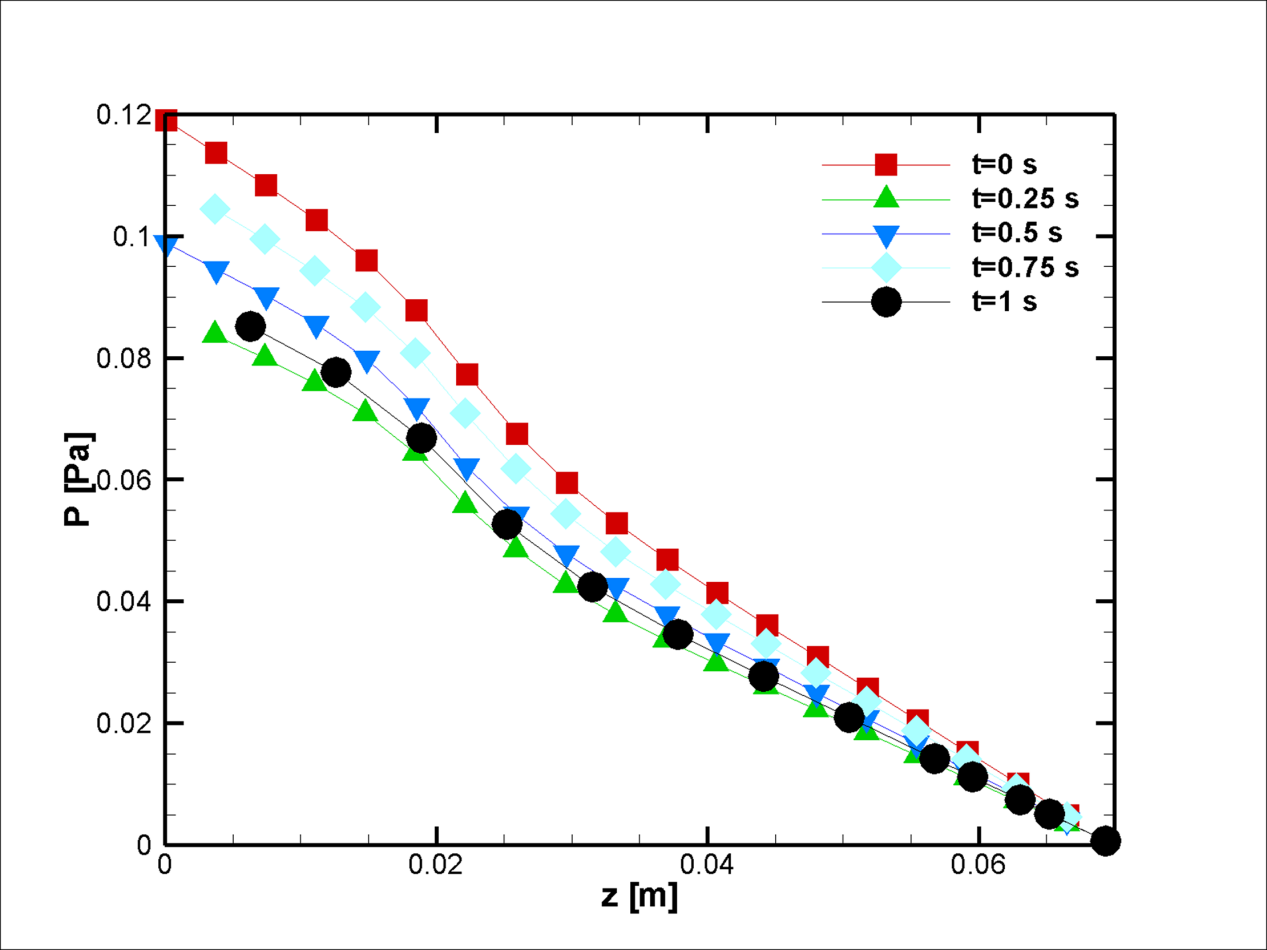
Pressure distribution along the mid-line of the stenosed (r = 2.5 mm) artery for the case without joule heating at five different time instances, *L*_*vessel*_ = 7 *cm*, *R*_*vessel*_ = 1 *cm* and fifty percent stenosis.

**Fig 11:**
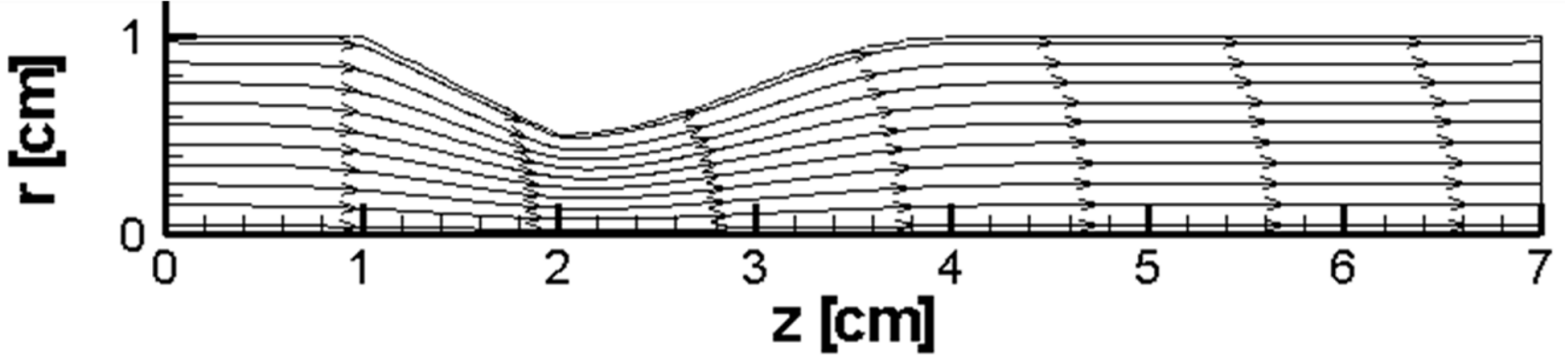
Streamlines for the case without Joule heating at t = 0.5 s, *L*_*vessel*_ = 7 *cm*, *R*_*vessel*_ = 1 *cm* and fifty percent stenosis.

**Fig 12:**
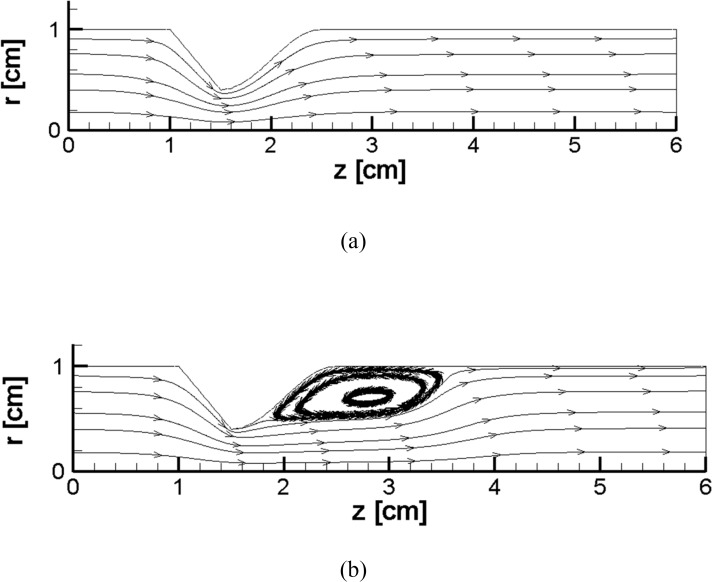
Comparison of the temperature fields at the middle of simulation (t = 0.5s) (a) the case with $\frac{r_{s}}{r_{in}}=0.5$ (b) the case with $\frac{r_{s}}{r_{in}}=0.6$.

The pressure distribution during transient simulation shows a similar pattern (Fig 10). The resulting velocity vector and streamlined distribution are plotted in Fig 11. The imposed magnetic field and the pressure difference change the temperature field (see Fig 12). Such observations are in concordance with the works of Sarifudiddin [64] and Pontrelli [65]. The intravascular pressure is of importance because it can be correlated with the interstitial fluid pressure (IFP) of a tumor [66]. In drug delivery applications of a tumor treatment, therapeutic agents should be injected at higher pressures to improve the method’s efficacy. The variation in temperature in Fig 12 is due to a variation of the velocity field that affects the convection of heat inside the blood vessel.

**Fig 13:**
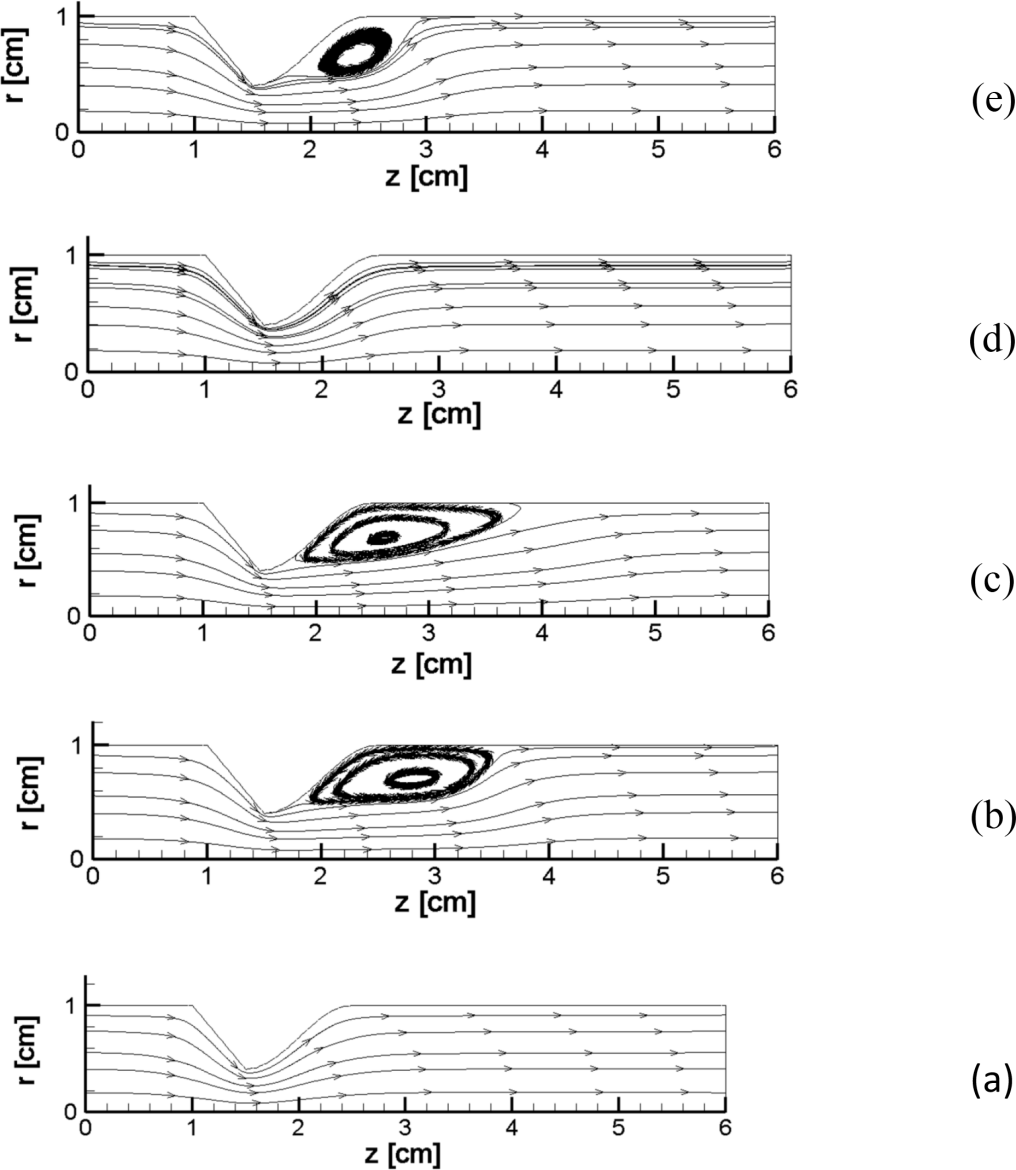
**(a) to (e).** This figure represents recirculation after the stenosis for the case of 60 percent stenosis with a low pressure gradient and Joule heating (a) t = 0 s (b) t = 0.25 s (c) t = 0.5 s (d) t = 0.75 s (e) t = 1 s, *L*_*vessel*_ = 6 *cm*, *R*_*vessel*_ = 1 *cm* and a fifty percent stenosis.

For smaller initial pressure differences in the vessel, even an area of recirculation is formed after the stenosis in comparison with a larger pressure difference (Figs 11 and 13). By an increase in the pressure difference, the recirculation zone is larger (see Fig 13A to 13E). Similar behavior is also present in literature [59, 67].

As is obvious, the most of the heating is due to Joule heating. Moreover, the importance of considering this heating is evident in Fig 12A and 12B, where the amount of temperature increases by an increase in the magnetic field strength. Additionally, the heating zone becomes larger because the area under the influence of the magnetic field enlarges.

As an example, in Fig 13, five different streamlines during the simulation of the blood vessel are presented. Initially, there is a smooth streamline; then, a recirculation area appears. The wake enlargement with time is evident in the Figs, which agree well with findings in the studies that are already present in the literature ([64], [65] and [34]). In the sensed area, the wake enlargement occurs because of vortex generation in the stenosed wall.

In all of the time instants, regions that have the highest amount of viscosity are limited to the axis of the blood vessel or the bio-fluid regions in the area with recirculation (Fig 13, no circulation in case e); at the same time, there is a reverse association between the gradients in the velocity field and the value of the viscosity. The viscosity of the bio-fluid stuck to the vessel wall far from the location of the stenosis is mostly low, and the viscosity of the bio-fluid is higher in the direction of the axis of symmetry. By means of the repulse of the vorticity at the location of the termination of the stenosis, the bio-fluid regions that contain the highest viscosities (and the highest velocity gradients) extend to the solid boundaries of the arterial blood vessel and eventually move the symmetry axis sooner (not far away from the symmetric line in Fig 13).

**Fig 14:**
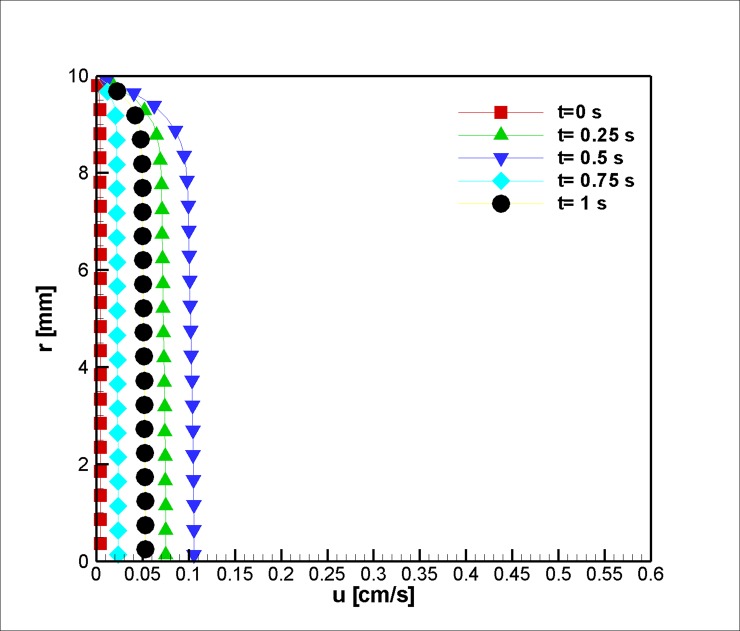
Velocity in the axial direction before stenosis at the inlet at z = 2 mm for the case of Joule heating, *L*_*vessel*_ = 7 *cm*, *R*_*vessel*_ = 1 *cm* and a fifty percent stenosis.

**Fig 15:**
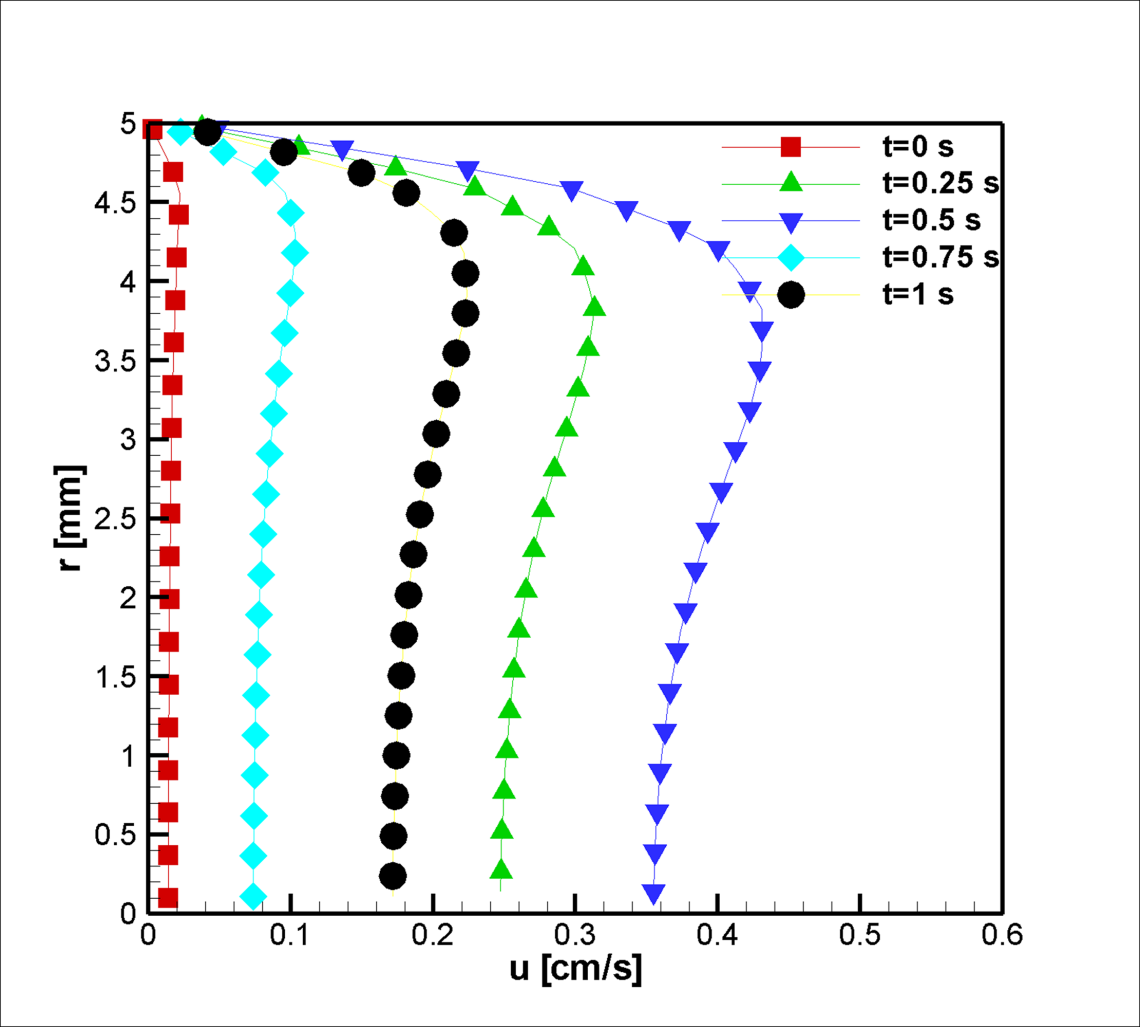
Velocity in the axial direction at the stenosis peak at z = 2 cm for the case of Joule heating, *L*_*vessel*_ = 7 *cm*, *R*_*vessel*_ = 1 *cm* and fifty percent stenosis.

**Fig 16:**
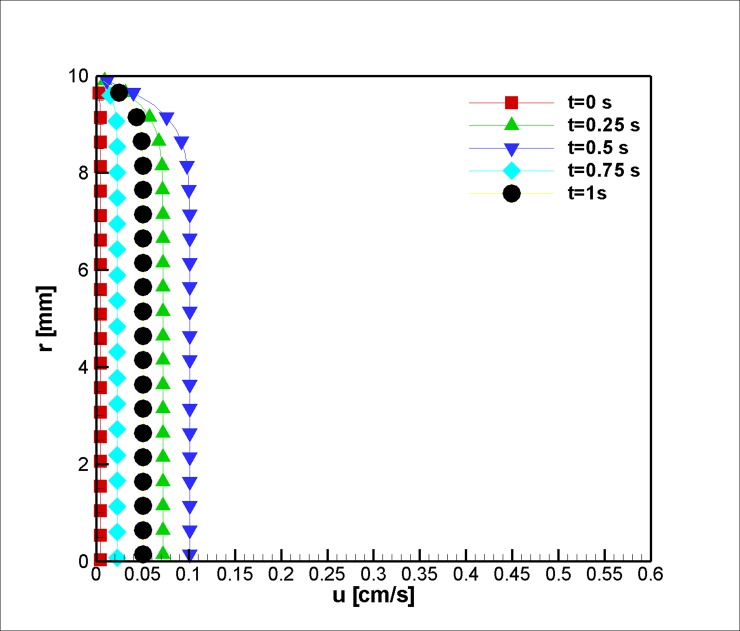
Velocity in axial direction at z = 4 cm for the case of Joule heating, *L*_*vessel*_ = 7 *cm*, *R*_*vessel*_ = 1 *cm* and fifty percent stenosis.

In the second case, flow in a tapered blood vessel under a constant magnetic field was studied, and the effects of the magnetic field on the velocity, before (z = 2 mm), at stenosis (z = 2 cm), and after it (z = 4 cm), are graphically presented and explained (Figs 14, 15 and 16). The graphs are also presented to visualize the temperature distribution in a blood vessel under the above-mentioned circumstances. Heat is removed from the second portion of the stenosis wall. Thus, the cooling effect reduces the harmful effects of the Joule heating on the blood, which can affect the Joule heating of the adjacent tissue.

**Fig 17:**
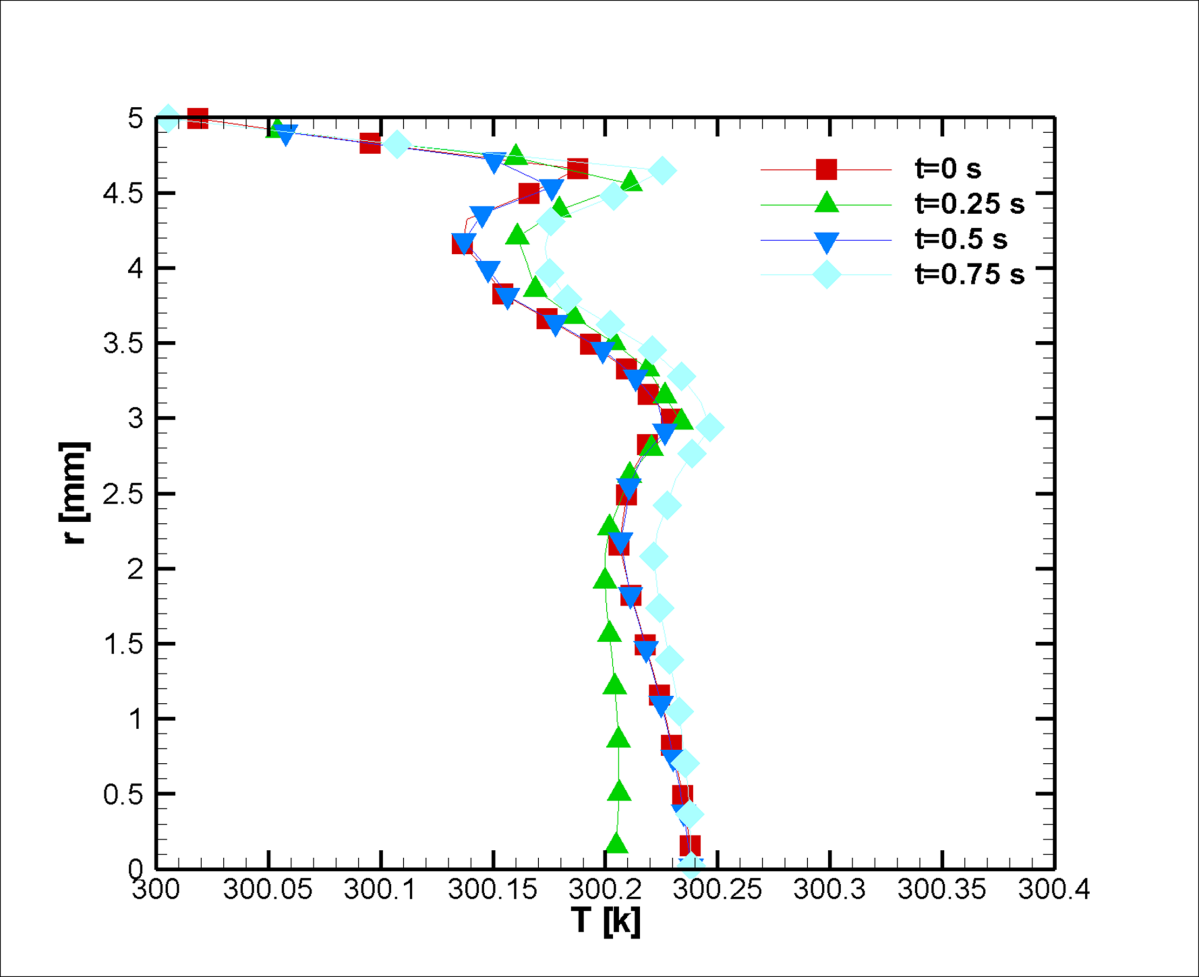
Velocity in the axial direction at z = 4 cm for the case of Joule heating, *L*_*vessel*_ = 7 *cm*, *R*_*vessel*_ = 1 *cm* and fifty percent stenosis.

**Fig 18:**
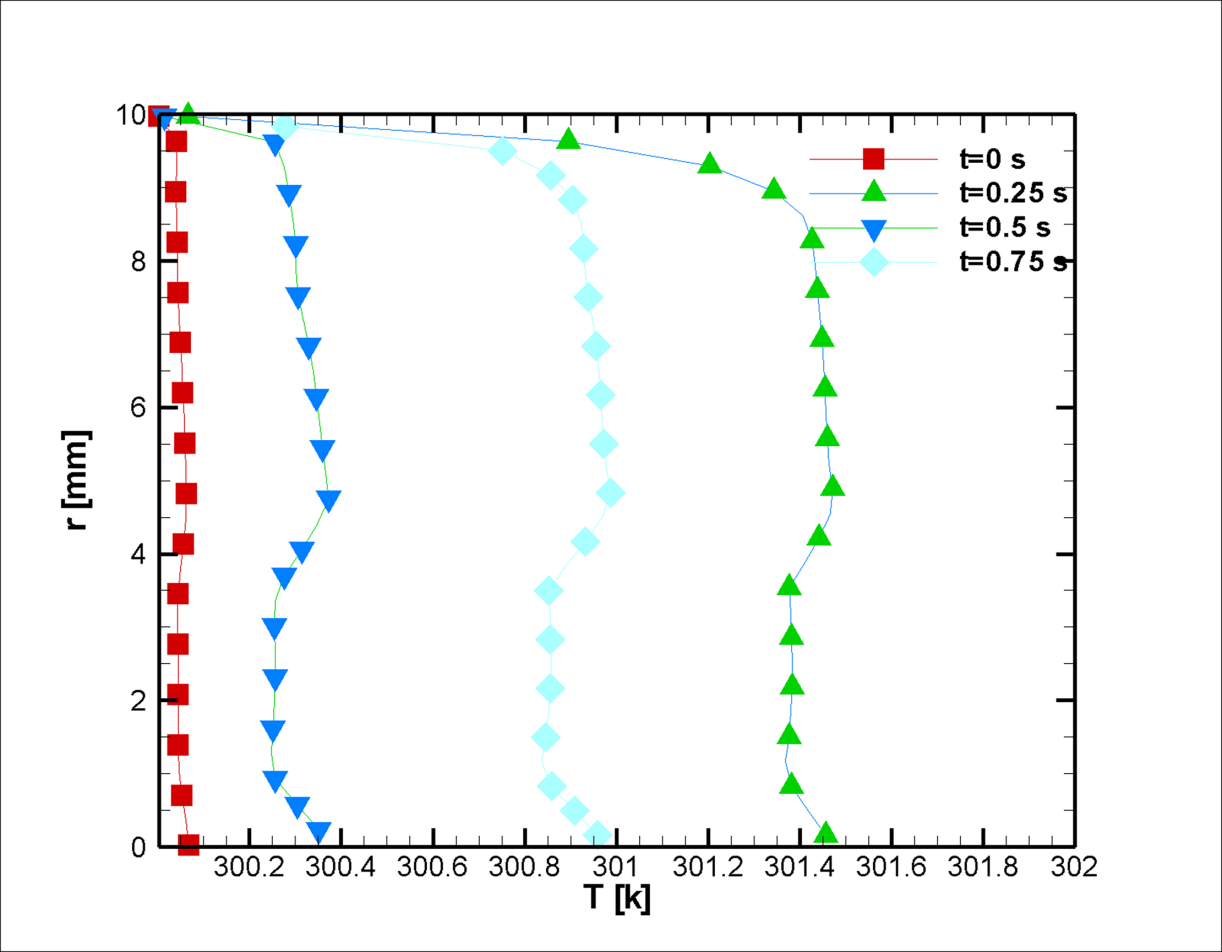
Temperature distribution at z = 4 cm for the case of Joule heating at various simulation instances, *L*_*vessel*_ = 7 *cm*, *R*_*vessel*_ = 1 *cm* and fifty percent stenosis.

In this case, in comparison with Fig 12 cases, the temperature varies less. However, the thickness of the thermal boundary layer occupies almost half of the vessel; however, the temperature fluctuation is rather small and negligible (Figs 17 and 18), since this amount of heat (nearly 37°C) could be removed by blood perfusion.

**Fig 19:**
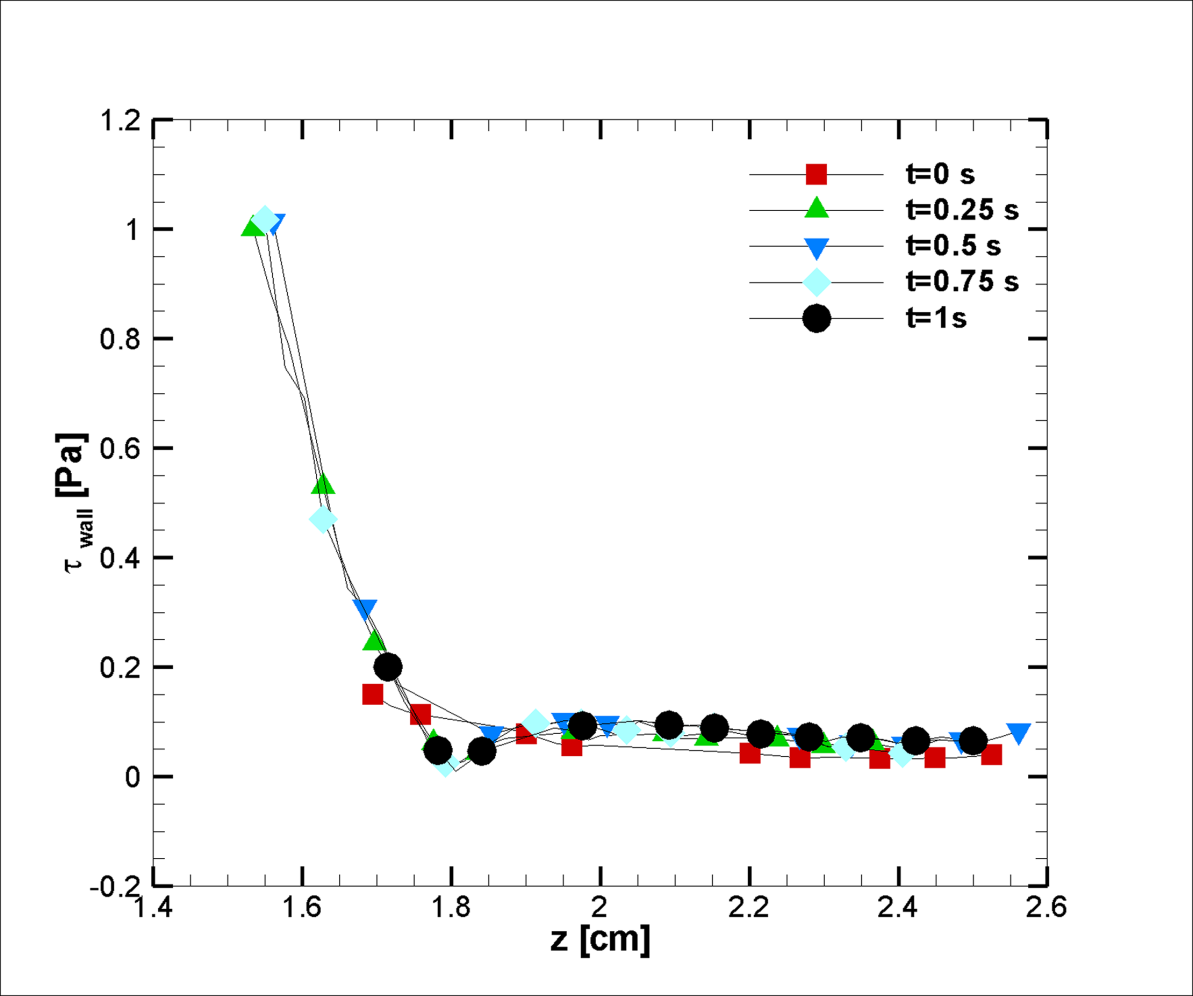
Wall shear stress for a case with blood recirculation ($\frac{r_{s}}{r_{i}}=0.6$).

As discussed earlier, for cases with a lower pressure gradient, circulation formed near the stenosis and after the stenosis peak. This phenomenon can lead to a plaque rupture and an embolism. Wall shear stress is a dominant factor in this regard. Additionally, good knowledge of skin friction leads to an understanding of the blood flow influence on the endothelial cells [68–70]. Thus, Fig 19 shows stress on the wall after the stenosis in the case of blood recirculation formation. Stress at the stenosis shows drastic drops after the stenosis peak and maintains an almost constant value with a slightly unsteady behavior; and because it changes with time, exerting a variant force on the vessel wall can cause faster plaque ruptures.

**Fig 20:**
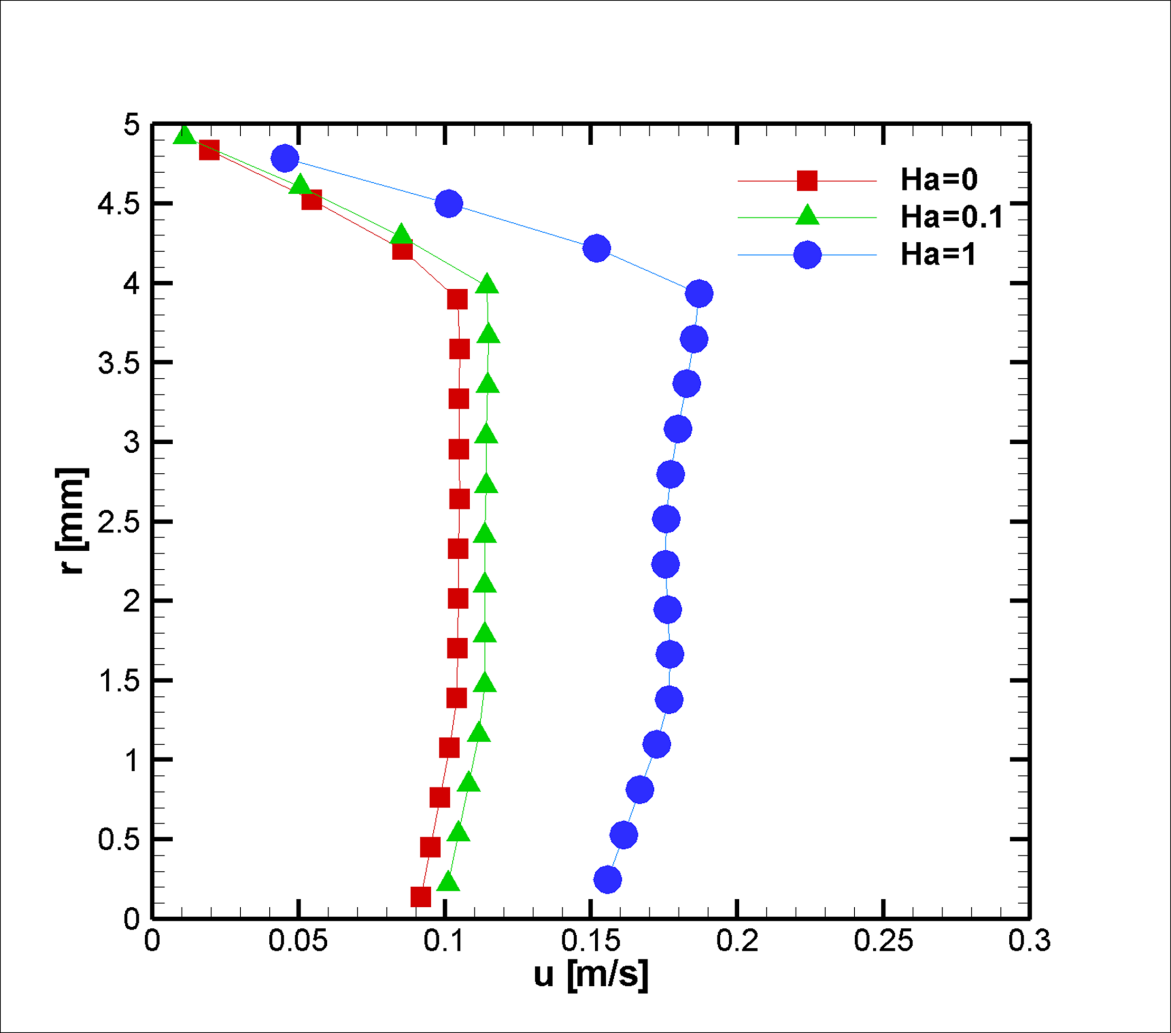
Velocity in the axial direction distribution for three different Ha Numbers at z = 2 cm and t = 0.5 s, *L*_*vessel*_ = 7 *cm*, *R*_*vessel*_ = 1 *cm* and a fifty percent stenosis.

**Fig 21:**
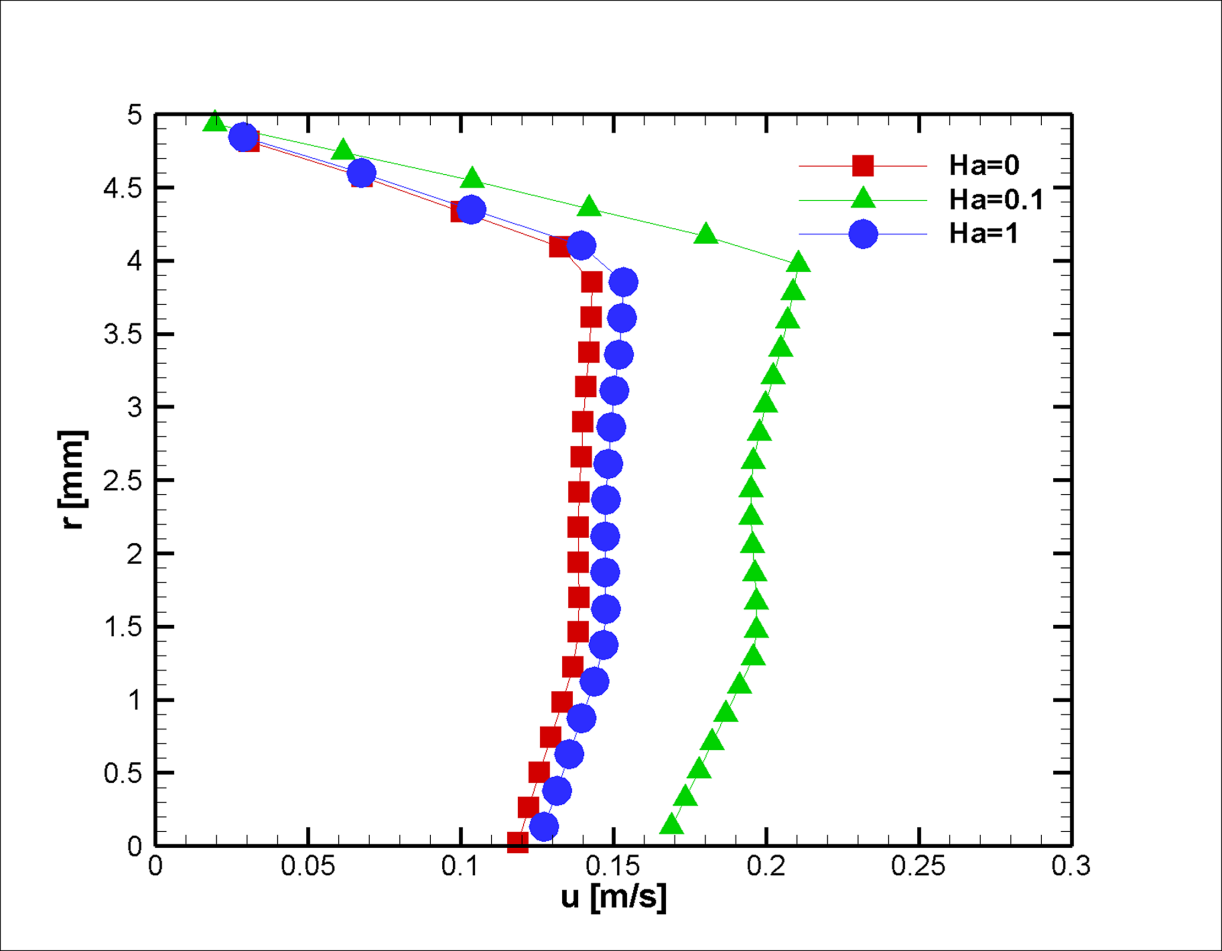
Velocity in the axial direction distribution for three different Ha Numbers at z = 2 cm and t = 0.75 s, *L*_*vessel*_ = 7 *cm*, *R*_*vessel*_ = 1 *cm* and fifty percent stenosis.

**Fig 22:**
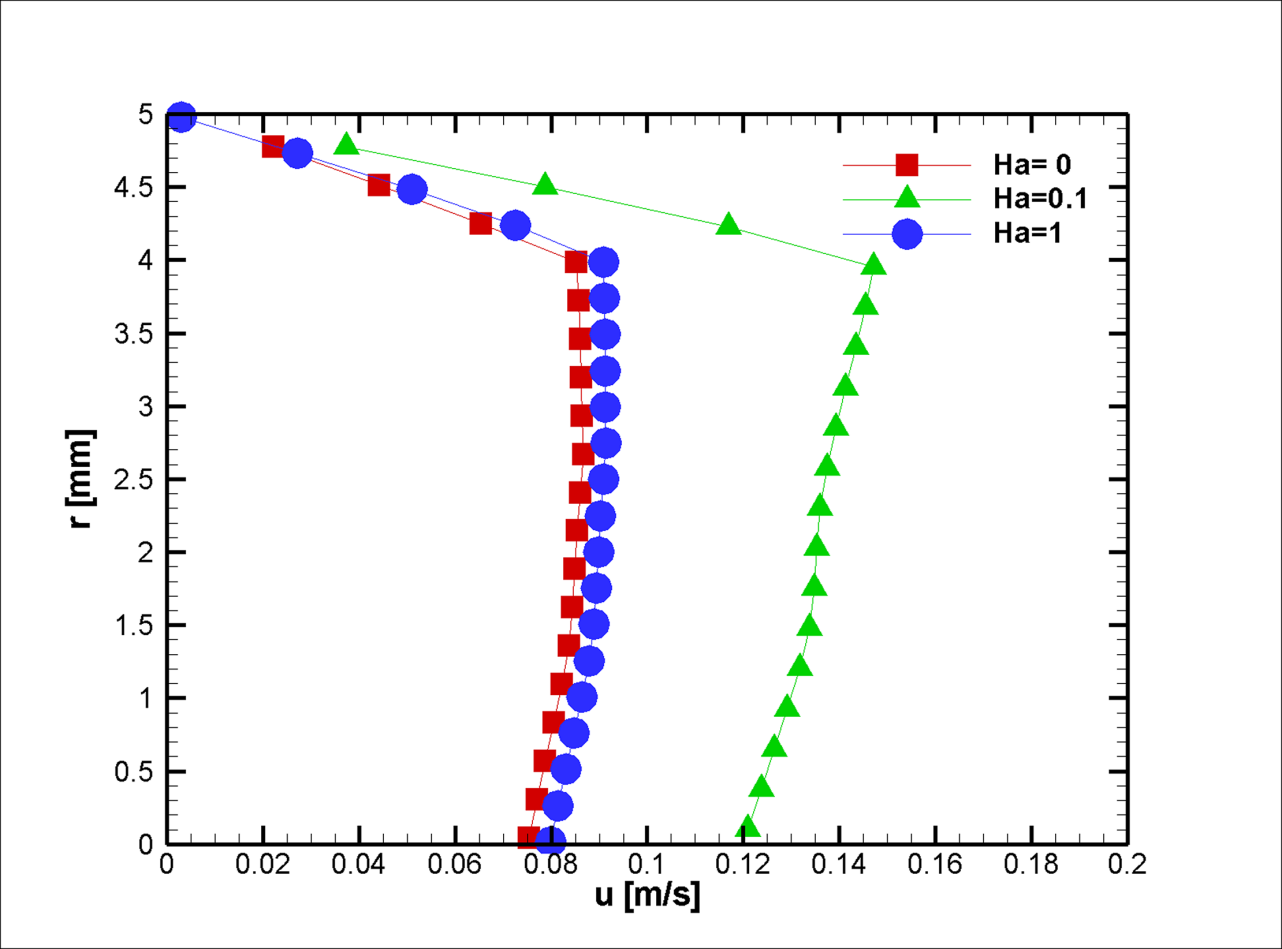
Velocity in axial direction distribution for three different Ha Number at z = 2 cm at t = 0.75 s, *L*_*vessel*_ = 7 *cm*, *R*_*vessel*_ = 1 *cm* and fifty percent stenosis.

The ratio of the electromagnetic body force to the effective viscous body force is presented by the Hartmann number. To elucidate the effect of different Hartmann numbers on the velocity field, three different applied magnetic fields (0, 0.1, and 1 Hartmann Numbers) were exposed on the blood vessel, and the velocity profiles are presented in Figs 20–22. Based on the results above, one can interpret the linear increase in the velocity with each incremental increase in the HA number. With regard to the magnetization limit, magnetic tests on humans were conducted in literature up to 8 T and on animals up to 16 T, and the subjects survived [71].

**Fig 23:**
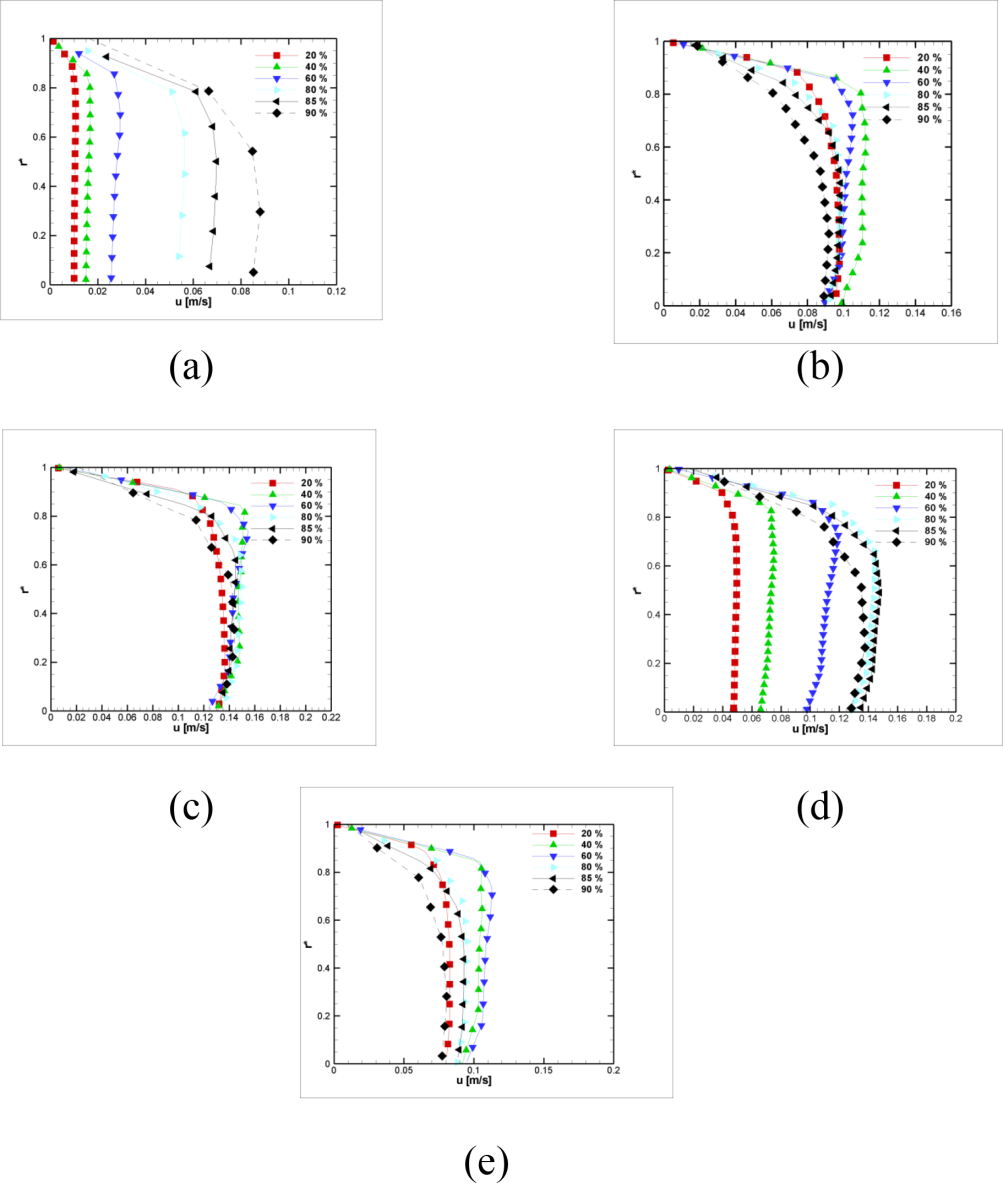
Velocity in the axial direction distribution for six different stenosis blockage percent peaks at z = 2 cm (a) t = 0 s (b) t = 0.25 s (c) t = 0.5 s (d) t = 0.75 (e) t = 1 s, ***L***_***vessel***_ **= 6 *cm*, *R***_***vessel***_ **= 1 *cm***.

Usually, the stenosis blockage augmentation increases the flow pressure drop. However, the difference between different stenosed geometries vanishes at the middle of the simulation (Fig 23(C)) and reappears afterward (Fig 23(D)). On the other hand, at *t* = 0.75 *s*, the pressure drop relation with the blockage reverses, and the lowest blockage imposes the highest pressure drop.

**Fig 24:**
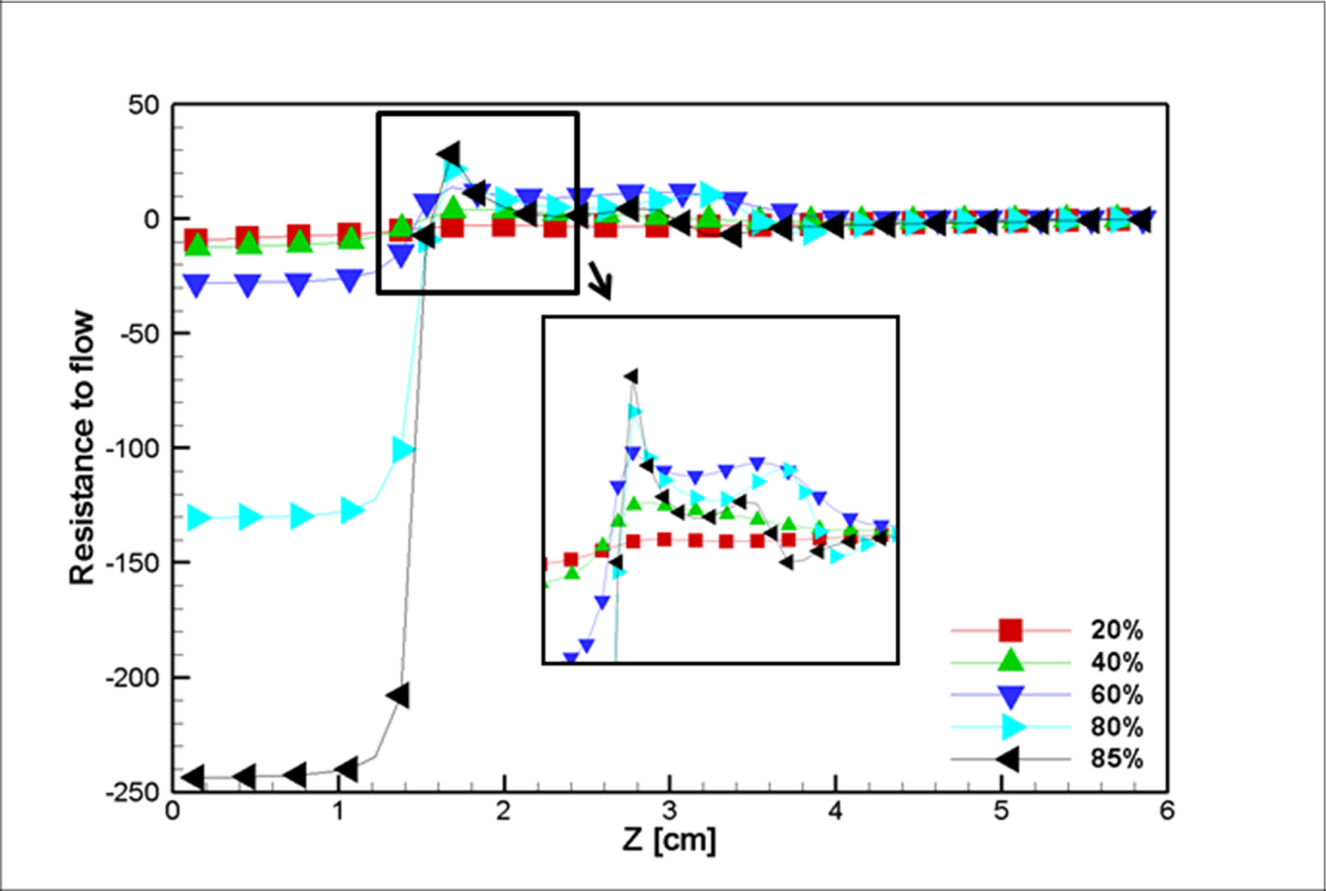
Resistance to the flow for five different stenosis blockage percentages over the midline of the blocked vessel at the middle of the simulation, t = 0.5 s, *L*_*vessel*_ = 6 *cm*, *R*_*vessel*_ = 1 *cm*.

Fig 24 depicts the flow pressure drop for different stenosis percentages (which range from a very mild (20 percent) stenosis to a severe (85 percent) stenosis. The difference between the inlet and outlet pressure drop in the vessels with a 60 percent (or less) blockage is negligible compared to larger blockages, such as an 80 percent blockage. For example, with an increase from a 60 percent to 80 percent blockage (a 1.41X enlargement of the stenosis), the pressure drop increases by approximately 10X. It is worth noticing that the work present in literature considered milder stenosis (i.e. up to 70 percent) and suggested the stenosis depths over fifty percent would change the resistance enormously [72], however the magnetization increased the flow resistance in our research which agrees with the findings of Xenos [73]. The large pressure drop in these cases can be correlated to the size of the trailing eddy after the stenosis. This eddy occupies a large area of the vessel in these cases. Moreover, the resistance curve follows an almost steady behavior after the stenosis peak. This behavior is because blood requires a greater amount of pressure difference to flow from the small narrowing of the blockage.

**Fig 25:**
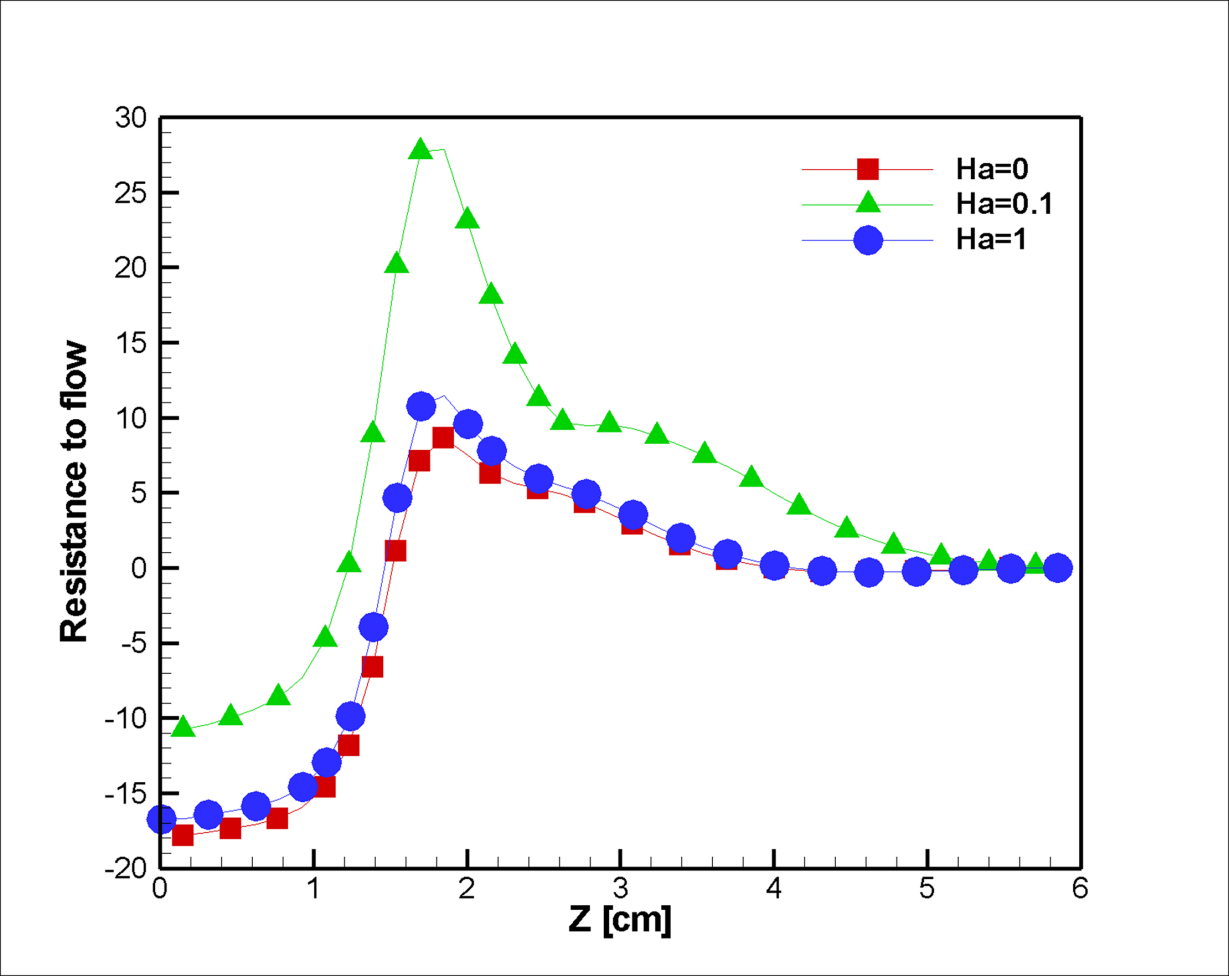
Resistance to the flow for five different stenosis blockage percentages over the midline (r = 1.25 mm) of the blocked vessel at the middle of the simulation, t = 0.5 s, *L*_*vessel*_ = 6 *cm*, *R*_*vessel*_ = 1 *cm*.

The pressure drop is also examined for the variation in the magnetic intensity in Fig 25. Increasing the magnetic field shrinks the vortex after the stenosis (the same trend is observed in the work of Xenos and Tzirtzilakis [73] for Newtonian flow), which reduces the resistance to the flow; however, the shear-thinning characteristics of the non-Newtonian fluid make the vortex size alteration less severe.

**Fig 26:**
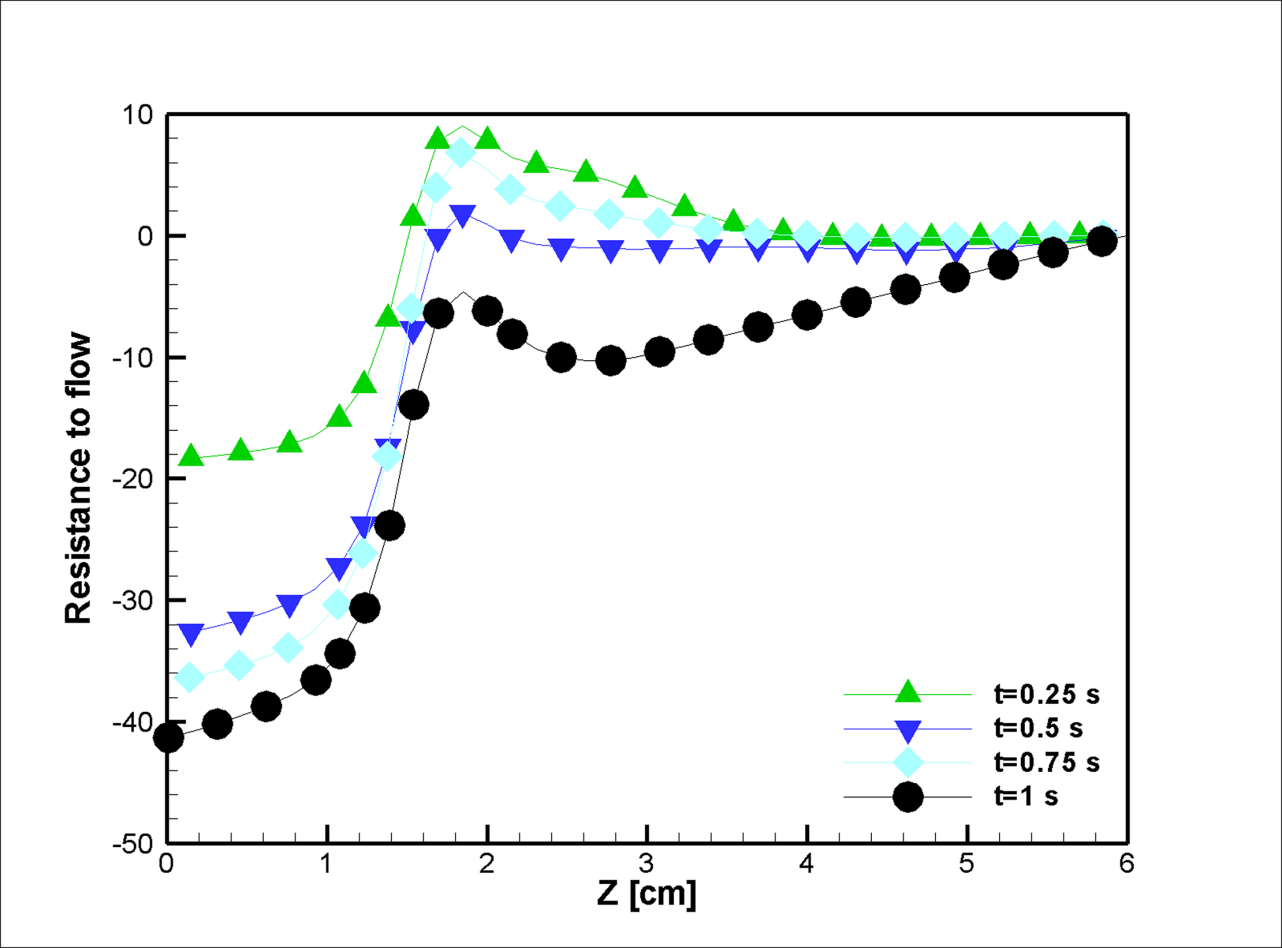
Variation in the resistance to the flow along the midline of stenosis (r = 2.5 mm) for different time instances in the case without joule heating. (***L***_***vessel***_ **= 6 *cm*, *R***_***vessel***_ **= 1 *cm***, 50 percent stenosis).

**Fig 27:**
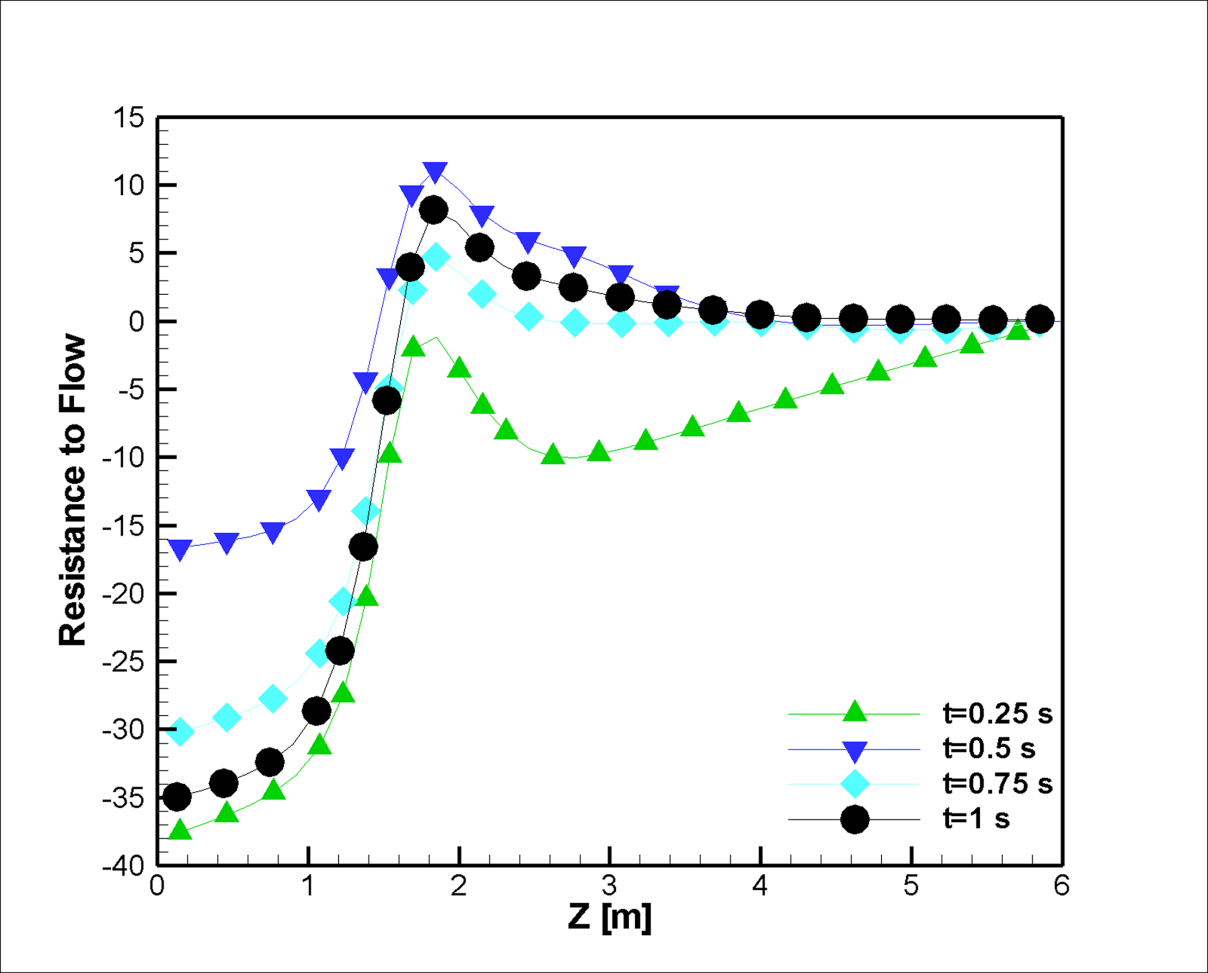
Variation in the resistance to the flow along the midline of the stenosis (r = 2.5 mm) for different time instances in the case with joule heating. (***L***_***vessel***_ **= 6 *cm*, *R***_***vessel***_ **= 1 *cm***, 50 percent stenosis).

Figs 26 and 27 represent the resistance to the flow in different time steps. Usually, exertion of magnetic field on the blood vessel diminishes the variation in the pressure drop due to the reduction of the blood recirculation and consequently eases the flow movement, which makes the pressure difference smaller. Additionally, the velocity increases at t = 0.25 with an augmentation of the magnetization, which is another reason for the reduction in the pressure at this time step.

## Conclusions

An unsteady axisymmetric code for the simulation of non-Newtonian Carreau-Yasuda fluid has been developed for the stented artery tube. It is based on the SIMPLE method in a complex geometry that shows a high geometric gradient. Two cases are considered for the fluid flow investigations. Major differences are seen in the wall shear stresses. The non-Newtonian model has better prediction values for the wall shear stresses and temperature distributions, and the following results are obtained:

- The non-Newtonian pulsatile flow’s main characteristics are unchanged, which reinforces the maximum shear stress and the normal pressure at the solid wall, and there is a decrease in the magnitude of the bio-fluid vorticity as well as a decrease in the length of the recirculation zone and the bio-fluid zone detachment from the vessel wall.
- The bio-fluid rheology characteristics cause a sudden increase in the axial velocity (axial mass flow rate) and wall friction at the site of the stenosis.
- An appropriate magnetic field can be applied to manage and control the flow behavior of the blood.
- The blood temperature monitoring in the case of a tapered artery is of paramount importance to maintaining the blood temperature in the range of living conditions.

These investigations provide valuable information for medical practitioners who seek to understand the flow of blood under stenosis conditions as well as for treating hypertension patients using magnetic therapy.
